# Mass animal sacrifice at casas del Turuñuelo (Guareña, Spain): A unique Tartessian (Iron Age) site in the southwest of the Iberian Peninsula

**DOI:** 10.1371/journal.pone.0293654

**Published:** 2023-11-22

**Authors:** Mª Pilar Iborra Eres, Silvia Albizuri, Mario Gutiérrez Rodríguez, Joaquín Jiménez Fragoso, Jaime Lira Garrido, María Martín Cuervo, Rafael M. Martínez Sánchez, Rafael Martínez Valle, Ana Isabel Mayoral Calzada, Ariadna Nieto Espinet, Esther Rodríguez González, Silvia Valenzuela-Lamas, Sebastián Celestino Pérez

**Affiliations:** 1 Sección de Arqueología, Instituto Valenciano de Conservación, Restauración e Investigación (IVCR+i), Valencia, Spain; 2 Departament de Història y Arqueología–SERP, Universitat de Barcelona, Institut de Arqueología (IAUB), Barcelona, Spain; 3 Instituto Universitario de Investigación en Arqueología Ibérica, Universidad de Jaén, Jaén, Spain; 4 Departamento de Medicina Animal (Área de Medicina y Cirugía Animal), Facultad de Veterinaria, Universidad de Extremadura, Cáceres, Spain; 5 Centre d’Anthropobiologie et de Génomique de Toulouse (CAGT), CNRS UMR5288, Université Paul Sabatier, Toulouse, France; 6 Centro Mixto UCM-ISCIII de Evolución y Comportamiento Humanos, Madrid, Spain; 7 Departamento de Medicina Animal (Area de Medicina y Cirugía Animal), Facultad de Veterinaria, Universidad de Extremadura, Cáceres, Spain; 8 Departamento de Historia (Área de Prehistoria), Facultad de Filosofía y Letras, Universidad de Córdoba, Cordoba, Spain; 9 Departamento de Medicina Animal (Área de Anatomía y Anatomía Patológica Comparada), Facultad de Veterinaria, Universidad de Extremadura, Cáceres, Spain; 10 Grup d’Investigació Prehistòrica (GIP), Departament de Geografia, Història i Història de l’Art, Universitat de Lleida, Lleida, Spain; 11 Consejo Superior de Investigaciones Científicas (CSIC-Junta de Extremadura), Instituto de Arqueología (IAM-CSIC), Badajoz, Spain; 12 Consejo Superior de Investigaciones Científicas (CSIC), Institució Milà i Fontanals, Archaeology of Social Dynamics, Barcelona, Spain; University of Cape Town Faculty of Health Sciences, SOUTH AFRICA

## Abstract

Zooarchaeological analyses of the skeletal remains of 52 animals unearthed in the courtyard of an Iron Age Tartessian building known as Casas del Turuñuelo (Badajoz, Spain) shed light on a massive sacrifice forming part of a series of rituals linked to the site’s last period of activity and final abandonment. The rites took place towards the end of the 5th century BCE when both the building (intentionally destroyed) and the sacrificed animals were intentionally buried under a tumulus 90 m in diameter and 6 m high. The main objective of the zooarchaeological and microstratigraphic analyses was to determine the phasing of the sacrificial depositions. Evidence gathered from taphonomic assessments and a series of radiocarbon datings indicate that the sacrifices fall into three consecutive phases spanning several years. The findings of the zooarchaeological analyses clearly point to a selection of equid and cattle males. Adult equids predominate (MNI = 41) followed by adult and sub-adult cattle (MNI = 6). Pigs, in turn, are only represented by a few adults and sub-adult females (MNI = 4). Among the animals is a single dog of undetermined sex between 3 and 4 years of age. The fact that the animals are mostly adults discards the likelihood that they died from natural causes or an epidemic. In addition, the scenographic deposition of certain equids in pairs, as well as evidence of the burning of plant offerings, suggest an intentional ritualistic sacrifice. Nine of the initial depositions of Phase 1 in the SE quadrant were scattered and certain of their bones bear marks characteristic of both prolonged open air exposure and scavengers. Another 31 animals from Phases 1 and 2 are represented by almost complete, articulated skeletons, indicating they were promptly covered. Phase 3, by contrast, reveals both almost complete and partial animals bearing clear signs of processing for human consumption. This study thus sheds light on both the sequence of the animal sacrifices and the protocols linked to rites accompanied by the celebration of banquets. Certain features associated with the sealing of this building under a tumulus offer evidence of the decline of the Tartessian Culture. This study thus advances notions serving to contextualize ritual animal sacrifices in the framework of practice observed at other Iron Age sites in the Iberian Peninsula and elsewhere throughout Europe.

## 1. Introduction

Archaeological sites yielding evidence of large-scale animal sacrifices are unusual throughout the Mediterranean sphere during the Iron Age. Greek texts however occasionally refer to these features. They apply, for example, the term hecatomb when specifically alluding to the sacrifice of almost 100 oxen. They also at times allude to large numbers of animals, oxen or rams. However, they rarely cite equids, the animal most often represented at Casas del Turuñuelo, the site of this study. Greek sources also narrate the case of animal sacrifice in the Late Bronze Age. Book III of the *Odyssey* recounts the sacrifice of 81 black bulls by King Nestor on the beach of Pylos, whereas Book XXIII of the *Iliad* cites the sacrifice of 50 black rams by Peleus, the father of Achilles, if his son were to return alive from Troy. Another example is the offering of 100 head of cattle to Athena during the annual festival of Panathenaia [1]. The rare references to horse sacrifice appear in the framework of commemorations (Pausanias III 20,9; Polybius XII, 4b). Moreover, archaeological work has yielded a diversity of ritual equid sacrifices at sites throughout the Iron Age in the Iberian Peninsula [2–11]. The assemblage presented in this study consists of 52 animals deposited in a courtyard beside a monumental Tartessian building with two floors accessed by a staircase. It is unusual to both the Iron Age of the Iberian Peninsula and the Mediterranean area mainly due to the great number of equids, their sequence of deposition and their state of preservation.

The Tartessian Culture (9th-5th centuries BCE) presumably stemmed from the hybridization of Phoenicians arriving in the south of the Iberian Peninsula at the end of the 9th century BCE and the local population [12]. An initial phase labelled “Eastern Tartessian” (8th-7th century BCE) was confined to Iberia’s southern coastline while the “Middle Tartessian” (7th-6th century BCE), englobing the nucleus of Tartessos, developed throughout the Guadalquivir River Valley. The “Late Tartessian” (6th-5th century BCE), in turn, saw a spread northward to the Middle Valley of the Guadiana River. During this latest phase its original cultural nucleus along the Guadalquivir experienced a crisis (end of the 6th century BCE) which coincided with the development and a great period of splendor in territories beyond its original geographical scope [13], notably the Guadiana River Plain, the setting of Casas de Turuñuelo.

Casas del Turuñuelo in the Middle Guadiana Plain belongs to a restricted category of large Tartessian Iron Age constructions covered by tumuli, features that explain the site’s excellent state of preservation [14]. Only two were completely excavated: Cancho Roano (Zalamea de la Serena, Badajoz) [15] and La Mata (Campanario, Badajoz) [16]. The sanctuary of Cancho Roano (end of the 5th century BCE) also yielded evidence of horses and donkeys. Yet none are articulated and they only represent 25% (MNI = 13) of an assemblage dominated by caprines (sheep and goats), cattle and pigs. Moreover, contrary to Casas de Turuñuelo, the animals were recovered in a ditch. Cancho Roano only resembles Casas del Turuñuelo in that the animals are presumably linked to rites prior to the destruction and abandonment of a sanctuary [12]. La Mata, the second site, has even less parallels as it yielded no evidence of mass animal sacrifice. It is interpreted as an aristocratic residence tied to the storage of agricultural surplus [16].

The zooarchaeological findings of other Early Iron Age settlements in the southwest and south of the Iberian Peninsula suggest that domestic species such as cattle and caprines played a greater role than pigs, horses, donkeys and dogs. Research highlights two models as to caprine and cattle management. The first is that a greater focus was initially placed on herding caprines while the second saw the tendency change towards cattle. These patterns are recorded among both local settlements and the coastline colonies [17–32]. Despite this duality of exploiting one type of herd or another, the zooarchaeological data suggest that cattle were the most common sources of meat during the Early Iron Age. However, the transition to the Late Iron Age marked a break of this duality where the archaeozoological data [24] suggest an increase of caprines and pigs. In any case, a common feature to all detailed faunal records is the scarcity of equids. Although findings from the initial stages of the Iron Age do cite the presence of donkeys, in particular in Phoenician colonies, there is little evidence of their role in the diet of Iron Age populations. This relates to a change in the use of these animals evidenced by a differential treatment of the most common species in rites and burials compared to those unearthed in waste dumps [8, 33–36].

Although equid ritual sacrifices are identified at Iron Age sites beyond the Iberian Peninsula [37–40], zooarchaeologists until now have never identified evidence in this timeframe of the sacrifice of such large assemblage comparable to the hecatombs described in ancient texts. Casas del Turuñuelo thus sheds new light on the ritual sacrifice of equids during the Iron Age.

The current state of knowledge on mass animal sacrifice during the European Iron Age raises several issues. There is gap between the information offered by classical sources and the archaeological record as to the number, species and sex of the sacrifices, as well as the protocols of the rites. This renders it difficult to apply the Greek concept of “hecatomb” to western European Iron Age sacrificial contexts. There is nonetheless enough zooarchaeological data allowing to grasp of the preferences in terms of species, age and sex of the animals sacrificed across Europe during the Iron Age [1, 39, 41–43]. This data also serves in determining both the sequence of the events and which are the most suitable methods to approach the rituals behind the practices of sacrifice.

Along these lines, this study explores the uniqueness of the Casas de Turuñuelo mass sacrifice. Given the great number of the slaughtered animals in the courtyard (MNI = 52), many questions remain open. Was the sacrifice a single event? Can it be defined as a hecatomb? What are the species, ages and sex of the animals? Is the sacrifice linked to any known Tartessian rite? Is there a connection between the sequence of this sacrifice and the site’s monumental building and with other Tartessian sites in the Guadiana Plain covered by tumuli? These questions will be addressed through zooarchaeological, taphonomic and microstratigraphic analyses of a faunal assemblage from the courtyard of the building of Casas del Turuñuelo. Radiocarbon dating was applied to assist in determining the phasing of the different depositions and to reconstruct the actions linked to the rituals that ended with the sealing of the site under an earthen mound.

Initially the archaeozoological study of the bones was specifically to determine and quantify the different anatomical elements. This was followed by identifying their taxon, age, sex, morphological characteristics and potential pathologies. To identify the origin of the taphocoenosis, the study also explored the taphonomic processes through biostratinomic and fossildiagenetic approaches.

Microstratigraphic analyses, and more specifically archaeological soil and sediment micromorphology, also served to correlate the faunistic assemblage with the site’s natural and anthropogenic processes. Microfacies analyses in fact played a decisive role in determining the sequence of the sacrifices, how the bones were deposited and the postdepositional events affecting them. The combination of the results of these different disciplines thus shed new light on Tartessian rituals and the role of mass animal sacrifices in the framework of European Iron Age societies at the twilight of Iberia’s Tartessian Culture.

## 2. The site

**Fig 1:**
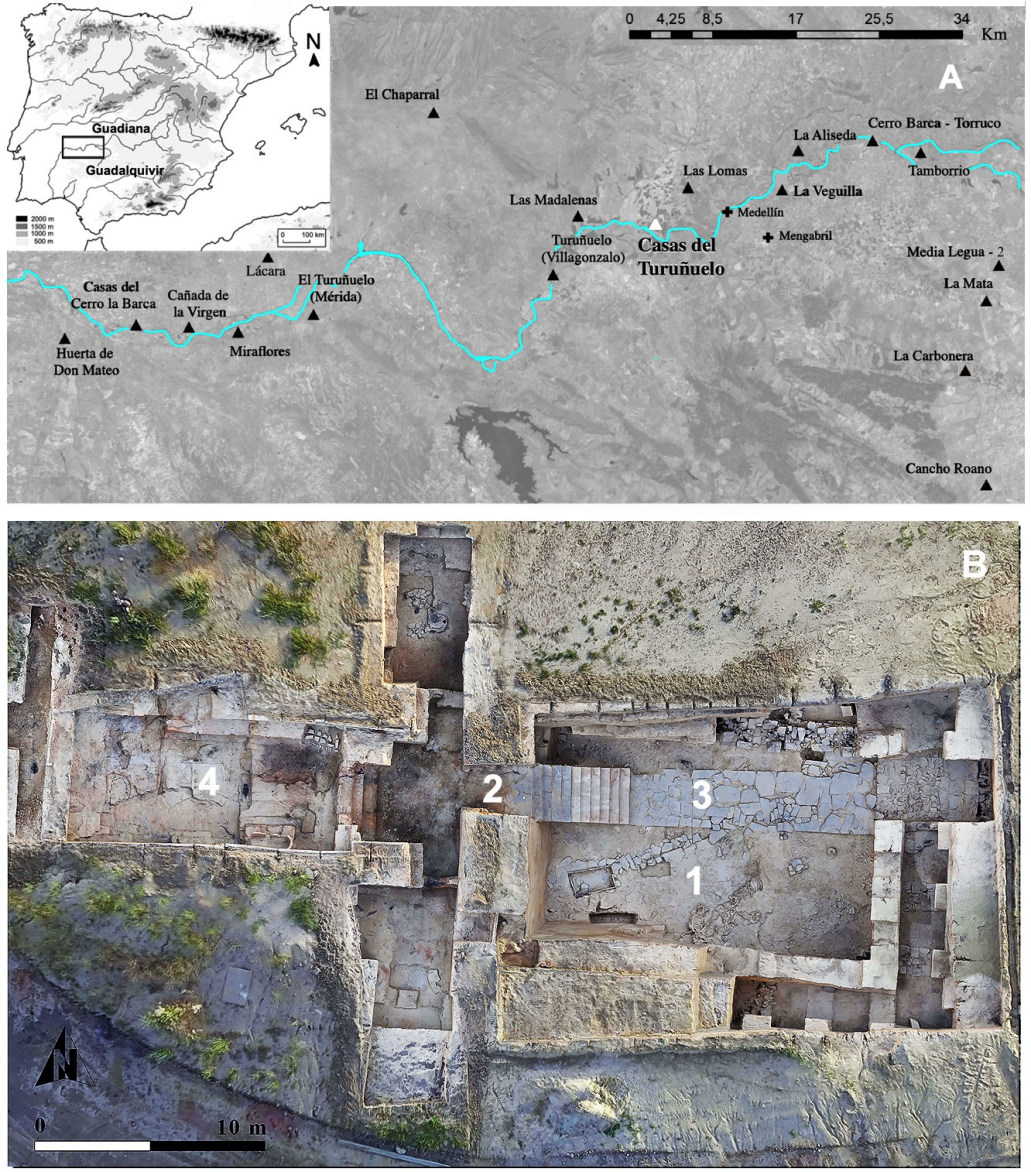
A. Map indicating the location of Casas del Turuñuelo (Guareña, Badajoz, Spain) and other archaeological sites in the Middle Guadiana River Plain (image from [46]). B. Aerial view of the monumental building, July 2021 (https://construyendotarteso.com/es/paginasITM/hecatombe-animal): 1 courtyard; 2 staircase; 3 slate paving; 4 upper floor main room.

Archaeological work at the site of Casas del Turuñuelo began in 2015 (Fig 1A). Four campaigns brought to light a well preserved large adobe Tartessian building that was party destroyed and covered by a tumulus towards the end of the 5th century BCE [44]. Two of its floors adjacent to an open courtyard (125 m2) containing the sacrifice are still standing [45].

**Fig 2:**
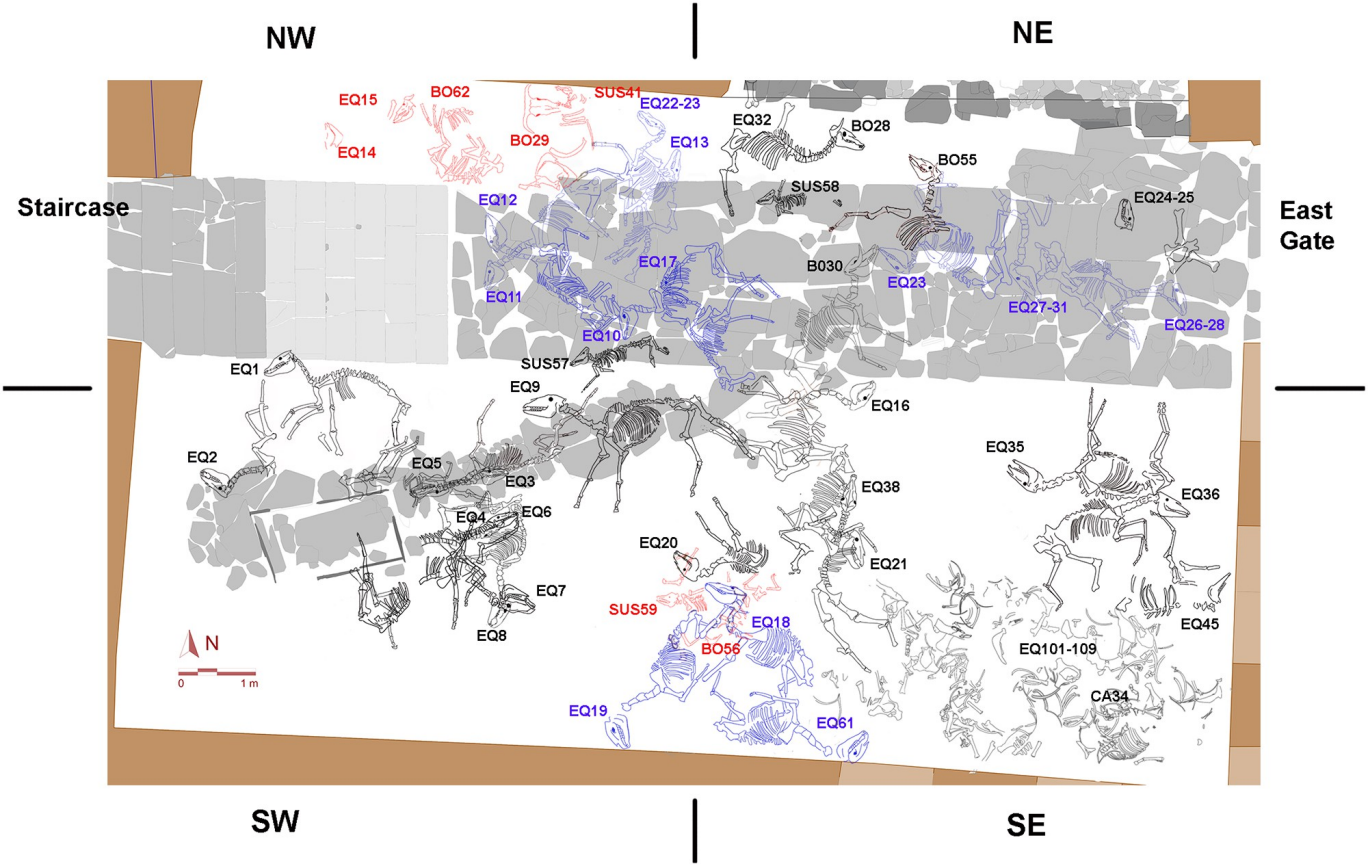
Plan of the courtyard of Casas de Turuñuelo, July 2021 (https://construyendotarteso.com/es/paginasITM/hecatombe-animal) indicating the animal depositions of Phases 1 (black), 2 (blue), and 3 (red). For the purposes of the excavation the surface of the courtyard was divided into 4 quadrants.

The excavation focused on three rooms of the upper floor, including the main room (Fig 1B) extending over a surface of 70 m2. The central axis of this main room features an oxhide-shaped clay altar, a structure characteristic both of Tartessian sanctuaries of the southwest of Iberia and temples and sanctuaries throughout the eastern Mediterranean [45]. The vestibule of the upper floor and the courtyard are bridged by a monumental staircase 3 m high (Fig 1B). The courtyard is so far the largest space associated with the building [47]. Its uniqueness is not only due to its dimensions, but to the presence of 52 domestic animal skeletons, mostly equids (Fig 2). Worth highlighting are those deposited on a corridor of slate slabs running through the courtyard connecting the base of the staircase (to the west) with the main access (to the east).

Other types of artifacts were likewise recovered in the courtyard the first, to the north of the staircase, consists of a set seven bronze weights (forming part of a single system) together with a fragment of wool, the oldest recorded to date in the Iberian Peninsula [48]. Other finds point to contacts with the Eastern Mediterranean. These include three Punic ointment jars made of vitreous paste (S1A Fig) and five sheep astragali bones (S1D Fig) (4 from the left foot; 1 from the right) bearing lateral and medial facets altered by abrasion. Furthermore, other import adding to the uniqueness of the site include traces of three bowls of Macedonian origin (S1B Fig) and a fragment of a sculpture of Pentelic marble from Athens (S1C Fig) recovered at the foot of the staircase [47].

## 3. Materials and methods

The 6770 bones collected from the three phases of sacrifice in the courtyard correspond to a minimum number (MNI) of 52 animals, consisting especially of equids (*Equus ferus caballus*, *Equus africanus asinus and Equus* sp.) followed by smaller numbers of cattle (*Bos primigenius taurus*), pigs (*Sus scrofa domesticus*) and a single dog (*Canis lupus familiaris*) (Table 1, S1A Table). They appear in form of several deliberate accumulations and articulated bone groups (ABG) [41, 49] consisting respectively of either isolated or partial bones or largely complete articulated skeletons. The assemblage comprises 34 almost complete, articulated individuals missing only a few bones that are assumed to be burned. However, eight skeletons are only represented by less than 50% of their bones, some of which were articulated. Finally, isolated bones (NISP = 400) correspond to 10 animals from the SE quadrant, notably 9 equids and 1 dog (Table 2). The taphonomic markers suggest that the low anatomical representation and dispersion of the bones in the southeast quadrant are mainly due to scavengers and anthropogenic activity subsequent to originally being deposited in whole.

**Table 1:** Description of the minimum number of individuals (MNI) recovered in the courtyard. Age in years and months (m). Sex M (male); F (female). Location: For the purposes of the excavation, the courtyard was divided into four quadrants, northwest (NW), northeast (NE), southwest (SW), southeast (SE), and the central paved slate corridor (SP). Anatomical representation: Complete (C), partial (P), isolated (IB) bones. Articulation: Articulation (A), Partially articulated (PA), Non articulated (NA). Laterality concerns the side on which the animal was lying: right (R), left (L), ventral (V). The asterisk (*) signals the cases where the primary position was disturbed by the collapse of the walls of the building. The thermoalteration column indicates the percentage of burned bones with respect to the total number of bones of each species. Others columns shows modifications (weathering, canid tooth marks, bird scavenging marks and butchery marks observed. For the individuals differentiated in the SE quadrant these modifications are expressed in Table 2.

sample	Taxon	Age	Sex	Phase	Location	Anatomical representation	Articulation	Laterality	Thermoalteration	Weathering	Carnivore tooth marks	Bird scavenging marks	Cut marks	Tooth human marks
EQ1	*Equus* sp.	5–6	M	1	SW	C	A	R	>50%	-	-	-	-	-
EQ2	*Equus* sp.	>6	M	1	SW	C	A	L	100%	-	-	-	-	-
EQ3	*Equus* sp.	5–9	M	1	SW	C	A	ventral	<3%	-	-	-	-	-
EQ4	*E f*. *caballus*	5–9	M	1	SW	C	A	ventral	<3%	-	-	-	-	-
EQ5	*Equus* sp.	6–7	M	1	SW	C	A	L	<3%	-	-	-	-	-
EQ6	*Equus* sp.	5–6	M	1	SW	C	A	L	<3%	-	-	X	X	-
EQ7	*E f*. *caballus*	5–6	M	1	SW	C	A	R	50%	-	-	-	-	-
EQ8	*Equus* sp.	6–7	M	1	SW	C	A	L	<3%	-	-	-	-	-
EQ9	*E f*. *caballus*	5–7	M	1	SW	C	A	R	-	-	-	-	-	-
EQ16	*Equus* sp.	8–9	M	1	SE	C	A	R	-	-	-	-	-	-
EQ20	*E*.*a*. *asinus*	4–5	F	1	SW	C	A	L	-	-	-	-	-	-
EQ21	*E f*. *caballus*	7–8	M	1	SE	C	A	L	<3%	-	-	-	-	-
EQ24-25	*Equus* sp.	5–6		1	SP	P	PA	*	<3%	-	-	-	-	-
EQ32	*Equus* sp.	4–5		1	NE	P	A	R	-	-	-	-	-	-
EQ35	*Equus* sp.	6	M	1	SE	C	A	L	<3%	-	-	-	-	-
EQ36	*Equus* sp.	5–7	M	1	SE	C	A	L	-	-	-	-	-	-
EQ38	*Equus* sp.	7–9	M	1	SE	C	A	ventral	-	-	-	-	-	-
EQ45	*Equus* sp.	7–9	M	1	SE	C	A	R	-	-	-	-	-	-
BO28	*Bos p*. *taurus*	3,5	F	1	NW	C	A	L	-	X	-	-	-	-
BO30	*Bos p*. *taurus*	>6	M	1	SP	C	A	L	-		X	-	-	-
BO55	*Bos p*. *taurus*	3,5	M	1	NW	P	PA	R	<3%	X	-	-	-	-
SUS57	*Sus s*. *domesticus*	3–3,5	F	1	SP-SW	C	A	R	<3%	-	-	-	-	-
SUS58	*Sus s*. *domesticus*	3–3,5	F	1	NW	P	A	R	<3%	-	-	-	-	-
EQ101	*Equus* sp.	Adult		1	SE	IB	NA							
EQ102	*Equus* sp.	Adult		1	SE	IB	NA							
EQ103	*Equus* sp.	Adult		1	SE	IB	NA							
EQ104	*Equus* sp.	Adult		1	SE	IB	NA							
EQ105	*Equus* sp.	Adult		1	SE	IB	NA							
EQ106	*Equus* sp.	Adult		1	SE	IB	NA							
EQ107	*Equus* sp.	Adult		1	SE	IB	NA							
EQ108	*Equus* sp.	Adult		1	SE	IB	NA							
EQ109	*Equus* sp.	Adult		1	SE	IB	NA							
CA34	*Canis l*. *familiaris*	3–4		1	SE	IB	NA		-	-	-	-	-	-
EQ10	*E f*. *caballus*	5–6	M	2	SP	C	A	R	-	-	-	-	-	-
EQ11	*Equus* sp.	5–7	M	2	SP	C	A	R	100%	-	-	-	-	-
EQ12	*Equus* sp.	3–4	M	2	SP	C	A	ventral	100%	-	-	-	-	-
EQ13	*E f*. *caballus*	4–5	M	2	SP	C	A	R	<3%	-	-	-	-	-
EQ17	*E f*. *caballus*	7–9	M	2	SP	C	A	L	<3%	X	-	-	-	-
EQ18	*Equus* sp.	5–7	M	2	SW	C	A	R	-	-	-	-	-	-
EQ19	*Equus* sp.	7–9	M	2	SW	C	A	R	-	-	-	-	-	-
EQ 22/33	*E f*. *caballus*	7–9	M	2	SE	C	A	R	<3%	-	-	-	-	-
EQ23	*Equus* sp.	7–9	M	2	SP	C	A	L	<3%	-	-	-	-	-
EQ26-28	*Equus* sp.	5–7	M	2	SP	C	A	L	-	-	-	-	-	-
EQ27-31	*Equus* sp.	5–7	M	2	SP	C	A	ventral	<3%	X	-	-	-	-
EQ61	*Equus* sp.	5–7	M	2	SE	C	A	R	-	-	-	-	-	-
EQ14	*Equus* sp.	5–7	M	3	NW	P	A	*	100%	-	-	-	-	-
EQ15	*Equus* sp.	5–7	M	3	NW	P	A	*	100%	-	-	-	-	-
BO29	*Bos p*. *taurus*	>6	M	3	NW	C	PA	L	<3%	-	-	-	X	-
BO56	*Bos p*. *taurus*	5–6 m	M	3	SW	C	NA	V	<3%	-	-	-	X	X
BO62	*Bos p*. *taurus*	3,5	-	3	NW	P	PA	L	100%	-	-	-	-	-
SUS41	*Sus s*. *domesticus*	3	F	3	NW	P	NA	V	<3%	-	-	-	-	-
SUS59	*Sus s*. *domesticus*	2	F	3	SW	C	PA	ventral	-	-	-	-	-	-

**Table 2:** Anatomical representation of the animals in the SE quadrant of the courtyard identified by isolated bones and summary of the observed modifications in *equidae* bones.

	*Canis l*. *familiaris* (n = 1)	*Equus* sp.(n = 9)	Thermoalteration	Weathering	Carnivore tooth marks	Bird scavenging marks	Cut marks	Tooth human marks
skull	1	9	-	-	-	-	-	-
hyoid		2	-	-	-	-	-	-
mandibles	2	10	-	-	-	-	-	-
ribs		45	-	-	-	X	X	-
cervical vertebrae	6	18	-	-	-	-	-	-
thoracic vertebrae	3	56	-	-	-	X	-	-
lumbar vertebrae		5	-	-	-	-	-	-
sacrum		6	-	-	-	-	-	-
caudal vertebrae		13	-	-	-	-	-	-
scapula	2	10	-	-	-	-	-	-
humerus	1	10	-	-	X	-	-	-
ulna	1	9	-	-	-	-	-	-
radius	1	17	-	-	X	-	-	-
metacarpal	4	13	-	X	-	-	-	-
hipbones		15	-	-	X	-	-	-
femur		13	-	-	X	-	-	-
patella		8	-	-	-	-	-	-
tibia		10	-	-	-	-	-	-
carpus-tarsus and sesamoids	2	27	-	X	-	-	-	-
talus		6	-	X	-	-	-	-
calcaneus		8	-	X	-	-	-	-
metatarsal		9	-	X	-	-	-	-
Phalanx 1	4	17	-	-	-	-	-	-
Phalanx 2	2	19	-	-	-	-	-	-
Phalanx 3		16	-	-	-	-	-	-

### 3.1. Methods

Zooarchaeologists recovered and identified the bones. Anatomical and taxonomic identifications resorted both to the reference collections of Spanish institutions (Institut Valencià de Conservació, Restauració i Investigació (IVCR+i), Universidad de Córdoba (UCO) and Universidad de Extremadura (UEX) as well as atlases and specific studies of agriotypes and domestic forms [50–53]. Certain very small bones were possibly not identified as the sediment from the courtyard were not systematically sieved.

The total number of skeletal remains (NISP) served to quantify the relative abundance of each taxon. Skulls were counted as single remains. The minimum number of individuals (MNI) per taxon was easily estimated for the skeletons articulated. This number for the isolated bones was estimated by combining the best represented anatomical element, the laterality (right or left) and the state of epiphyseal fusion and dental eruption and wear [54, 55].

The biometric study in the case of equids followed the protocols outlined by Driesch [56] and Eisenmann [57].

The taxonomic identification of equids (*Equus ferus caballus*, *Equus africanus asinus*, *Equus* sp.) was complex due to the great morphological similarity between horse and donkey bones, as well as the diversity of morphological characters among the bones and teeth of their hybrids. This study therefore relied on published guidelines to distinguish the different species [51, 52, 58–63].

Age at death was estimated from patterns of incisor eruption and wear [64] as well as measurements of crown height and eruption-wear sequences [65]. Sex identification was initially based on the bone morphology and presence/absence of canines. When the canines were absent or residual, the animal was classified as female. When the canines were well developed, in turn, the animals were identified as male. The results on canines were contrasted with the morphology of the pubis [51] when it was well preserved. Withers heights were calculated according to the maximum length (GL) of metacarpal elements based on the factors proposed by May [66].

Determination of sex among cattle (*Bos primigenius taurus*) was carried out by analyzing the cranial anatomy, pelvic morphology (pubic bone) and metacarpal and metatarsal indices of robustness [67, 68]. The ages at death were differentiated based on the degree of bone fusion as well as the pattern of dental eruption and wear [69–71]. Estimates of height at the withers were calculated according to the Fock [72] and Matolcsi factors [73].

The specific determination and sex assigned to pigs (*Sus scrofa domesticus*) followed metric and morphological criteria based on the shape of the upper and lower canines and the pelvis [53, 74]. The age at death was set according to bone fusion and the degree of eruption and tooth wear [69, 70]. Withers height was determined according to the conversion factor of Teichert [75] and from the lengths of the radiuses, humeri, femurs, tibias and metacarpals III and IV.

Estimation of the age of the dog (*Canis lupus familiaris*) was based on tooth wear and epiphyseal fusion [51, 76]. Its withers height was estimated from data advanced by Harcourt [77].

The general interpretation of the faunal ensemble in the courtyard also stems from the study of the taphonomic alterations and the depositional sequence. The taphonomic effects were systematically studied through macroscopic observations and with a Zeiss Stemi 508 stereo microscope. Electron microscopy (SEM) was likewise put to use to record the micromorphological traces so as to determine their origin. The interpretation of butchery marks was based on the studies by Binford [78], Shipman and Rose [79], Pérez Ripoll [80] and Fernández Jalvo and Andrews [81]. Carnivore marks were identified according to the patterns reported by Binford [78], Haynes [82], Lyman [83], Yravedra et al. [84] and Indra et al. [85]. The traces linked to carrion-eating birds, in turn, were referenced by the works of Ballejo et al. [86], Domínguez-Solera and Domínguez-Rodrigo [87] and Reeves [88]. Human tooth puncture and scores were identified according to the patterns reported by Andrews and Fernández Jalvo [89], Martínez [90], Landt [91] and Saladié et al. [92].

Changes of color due to thermal alterations were defined based on the Munsell table. Those produced by the presence of manganese oxide dendrites, potentially associated with pedogenetic processes or humidity, were recorded according to the guidelines offered by Lyman [83]. Signs of exposure to environmental agents (often leading to cortical cracking of different form) and changes of surface coloration were recorded according to the criteria of Behrensmeyrer [93], Miller and Behrensmeyrer [94] and Nielsen-Marsh et al. [95].

Pathological alterations were likewise identified and described. These include trauma, malformations and infections of the bones and teeth based on the studies of Bendrey [96], Brown and Anthony [97] and Cook and Strasser [98].

The microstratigraphic analysis was carried out by means of archaeological soil and sediment micromorphology based on unaltered and oriented sediment samples collected from the chronostratigraphic profiles during the 2018 campaign. The sampling strategy was selective as it focused on sediments below and beside the skeletons of certain horses. The blocks of sediment were stabilized with plaster of Paris bandages before being oven-dried for one day at 50ºC. Impregnation with polyester resin (Palatal P4-01), styrene monomer and MEK catalyst was carried out under vacuum. This process yielded a total of 5 thin sections that were observed under plane-polarised (PPL), cross-polarised (XPL) and oblique incident (OIL) light following standard descriptive criteria [99–101]. The study here adhered to the microfacies concept by Flügel referring to arrangements of sedimentary elements into distinct and recurrent groups of similar composition and organization within a particular thin section [102]. These microfacies analyses led to grouping similar lithological groups, geometric associations and post-depositional alterations, thus helping identify recurrent patterns. This stems from the principle that distinct depositional environmental and post-depositional processes yield a particular set of microfacial units which are, in turn, linked to specific microfacies types [102, 103].

## 4. Results

### 4.1 Species

#### Equids

41 MNI (Table 1). This is the predominant animal in the courtyard. The bones morphometry and occlusal surfaces of their dentition suggest a majority of horses and a single donkey. Certain bearing traits of both horse and donkey are potentially hybrids (data to contrast with future genetic results in order to better hone the group).

Males dominate the assemblage (Table 1). This determination in the case of EQ4, initially based on its large canines, was recently confirmed by genetic analyses [104]. Their ages range for the most part from 5 to 7 years (55.27%), 4 to 5 years (15.79%) and older than 7 (28.95%). None surpasses the age of 10 (Table 1). Estimates of the height at the withers suggest a range between 125 and 141 cm (most around 135 cm). The withers height estimate for the sole donkey (EQ20) is around 110 cm (S1B Table).

Wear to the second premolar (PM2) is the most common pathology identified among the equids (except EQ20-*Equus a*. *asinus-*). Based on 23 of the 40 individuals subjected to this analysis (57.5%) (S2A Fig), the wear coincides with the position of metal bits. Twelve of the 40 cases (30%) also reveal a mandibular periostosis on the surface of the diastema (S2C Fig). This relates to friction and damage to the soft tissues produced by metal bit [61, 63]. This is confirmed by the presence of eight iron bits in the mouths of eight equids: EQ1 (S2B Fig), EQ3, EQ5, EQ7, EQ11, EQ13, EQ19 and EQ27/31.

The findings concerning the remaining bone structures indicate that more than half of the equids (22 out of 32 cases; 68.75%) present lesions of the thoracic vertebrae (S3 Fig), mainly exostoses caused by spondylosis. Another 33.3% reveal lesions to the metacarpal/metatarsal bones and primarily exostoses (11/33). Moreover, 30.3% of the horses show signs of osteoarthrosis in the proximal interphalangeal joints (10/33).

#### Cattle

6 MNI (Table 1). This is the second most common animal. The six identified in the courtyard correspond, according to the morphology of their pelvises, to four males and one female (one undetermined). The ages at death are less than six months (1), about 3.5 years (3), and more than six years (2). The height at the withers of the adult’s ranges from 119 to 125 cm (S1B Table). Pathological alterations are visible notably among the maxillae and mandibles of the occlusal surfaces and the vertical apophyses of the thoracic vertebrae of BO30 and BO29. There are also lateral deviations among the skeletons of BO55 and BO29. Finally, depressions are visible on the articular surface of the proximal epiphyses of the metacarpals of BO28 and BO55. These findings require further study to determine potential links between pathologies and the types of activities carried out by these animals.

#### Pigs

4 MNI (Table 1). This group comprises only four females, one of which was presumably pregnant judging by the fetal remains (a skull fragment, two humeri bones, and four ribs) unearthed in the NW quadrant next to SUS58. The age at death for one is two years, for another three years and for the last two 3–3.5 years. The heights at the withers are 69 cm for the youngest, 79 cm for the 3 year old and between 69 and 73 cm for the two older individuals (S1B Table). All were of fertile age and could have served as breeding females. They presented no significant pathology except one of the older individuals (SUS57 with the smallest molars) that suffered from an advanced case of dental hypoplasia (mainly the third molars) potentially resulting from an episode of metabolic or nutritional stress endured at about 12 months of age [105].

#### Dog

The last animal corresponds to a 3–4-year-old dog (Table 1) of undetermined sex. Its withers height ranges from 50 to 55 cm (S1B Table). An overgrown perforation of its palate indicates it survived an earlier trauma.

### 4.2 The sequence of depositions in the courtyard

Taphonomic, microstratigraphic and radiocarbon analyses served to reconstruct the rhythm of animal deposition in the courtyard of the monumental building (Table 3, S4 Fig). The sequence was also based on skeletal overlaps (Fig 2 and **Fig 9 in** S1 Appendix) and degrees of articulation. Identification of the marks and fractures served to interpret the agents leading to the displacement or absence of certain bones among both largely complete and partial skeletons. Observations of anatomical connections, as well as dispersion of isolated bones, also served to grasp the sequence of sediment accumulations and scavenging.

**Table 3:** Radiocarbon dating’s of the animal remains (Phases 1, 2 and 3). The calibrations were carried out with OxCal v4.4.4 software applying the IntCal20 calibration curve (Reimer et al. 2020) [106].

Animal	Element	Phase/Location	UCIAMS	14C age (BP)	±	Cal BCE 95.4% IntCal20
EQ35	M1 upper left	Phase 1-SE	260992	2420	15	717–409
BO30	Maxilla fragment.	Phase 1-SW	260997	2420	15	717–409
EQ13	M2 upper left	Phase 2-SP	260986	2400	15	537–403
EQ17	M2 lower right	Phase 2-SP	260987	2410	15	541–406
BO56	Maxilla fragment.	Phase 3-SW	260996	2355	15	455–390

In this regard, the traces of atmospheric agents suggest the carcasses were subjected to two main types of deposition. The first corresponds to those suffering from open air exposure for a period of time leading to carcass decomposition and disarticulation of the persistent joints, whereas the second type of deposition equates with those covered immediately, an action that diminished the effects of environmental agents.

Observation of thermoalterations, cut marks and fracture patterns also cast light on anthropogenic activities linked to the sacrifices. Furthermore, spatial analyses of the skeleton distribution, observations of degree of articulation and isolated bones also offered data on anthropogenic manipulations and repositioning of certain carcasses to make way for other depositions. These criteria in conjunction with the microstratigraphic analyses served to establish the rhythms of deposition which can be broken down into three distinct phases (S5 Fig).

#### Phase 0

The first archaeological level, prior to the deposition of any animal sacrifices, consists of a paving of a floor comprising river pebbles mixed with raw earth. This layer also includes 48 fragments of sheep, goat, cattle and pig bone. The dark tint of the surfaces of these bones stems from the precipitation of oxides. Furthermore, the fractures, thermoalterations and anthropogenic processing marks visible on these bones are characteristic of consumption prior to being disposed as waste.

**Fig 3:**
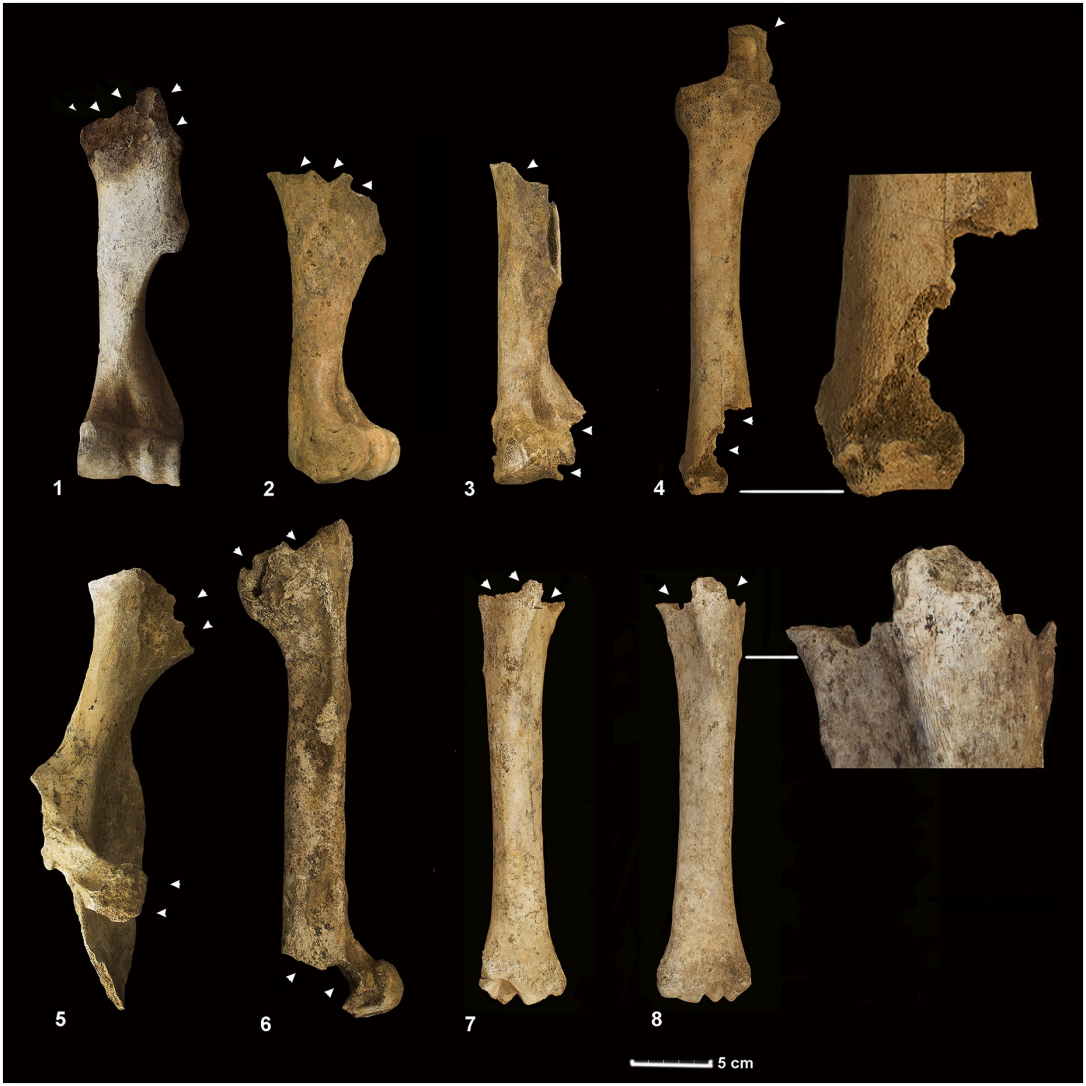
Bones bearing canid bite marks. Certain equids recovered in the SE quadrant reveal partial destruction of either their proximal epiphyses (1-2-7-8) or of both their proximal and distal epiphyses (3-4-6). They also exhibit partial destruction of the pelvises in the form of dentate fracture profiles at the ilium crest and the acetabular branch of the pubis (5). **1)** cranial side of the left humerus, **2)** lateral side of the right humerus, **3)** dorsal side of the left humerus, **4)** dorsal side of the left radius, **5)** medial side of the right pelvis, **6)** lateral side of the left femur, **7)** cranial side of the right tibia and **8)** cranial side of the left tibia.

**Fig 4:**
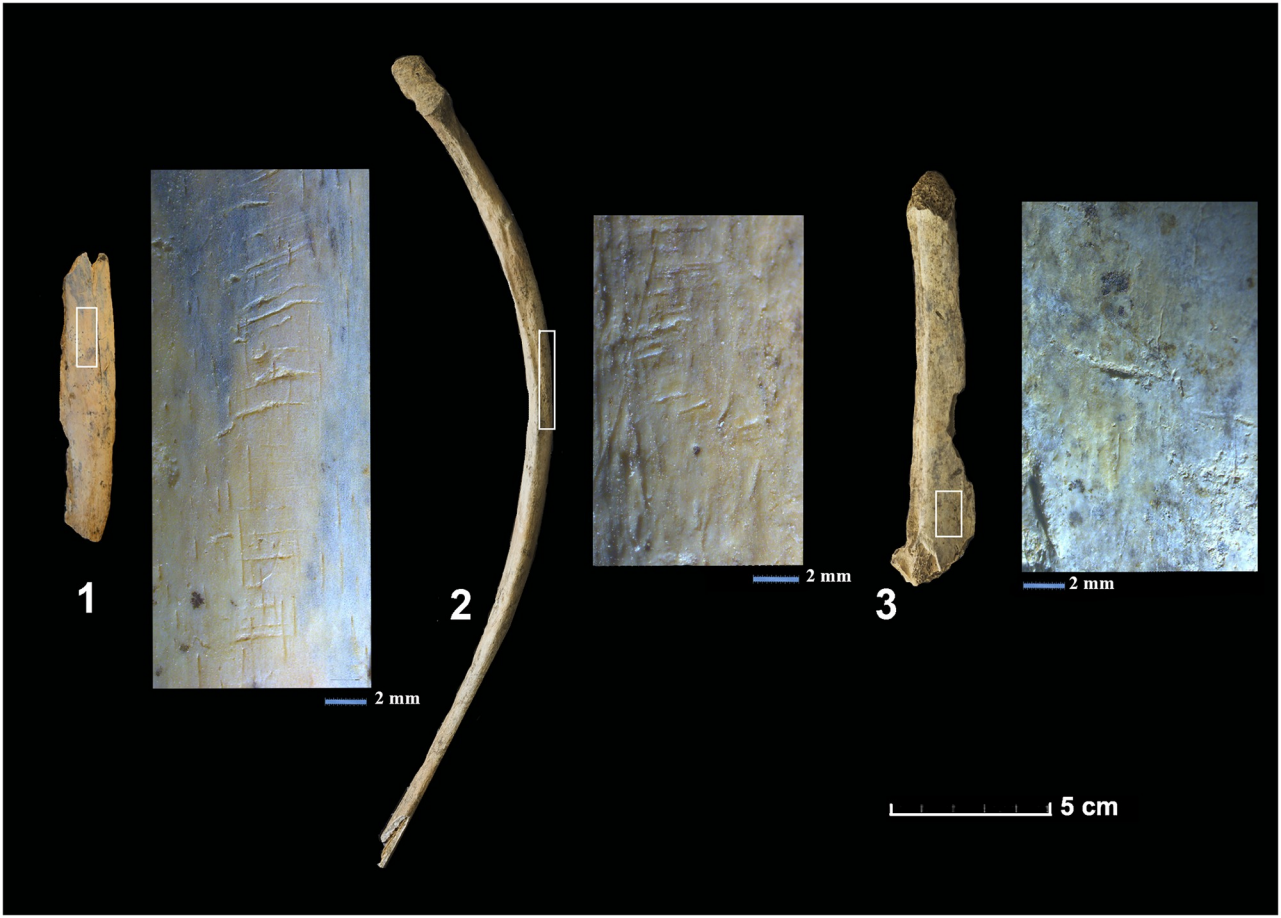
Bones bearing bird scavenging marks. Incisions and traces of dragging produced by the beaks of scavenging birds. **1)** lateral side of a rib, **2)** right rib caudal border, **3)** spinous process of thoracic vertebrae.

Microstratigraphic analyses offer a more detailed characterization of the initial layer. Its base consisted of quartz sand mixed with gravel (discoidal and rounded quartzites and slates), presumably bedload transported by the Guadiana River collected near the site. The upper beaten raw earth (preserved only near the monumental staircase), in turn, is characterized by pseudomorphic voids stemming from the decomposition of organic matter added as a binder (**Figs 3–4 in** S1 Appendix) [107].

#### Phase 1

This phase atop the pebble and beaten raw earth floor comprises most of the animal sacrifices. They consist of either largely complete or partial animals and isolated bones. These 33 animals, mostly males, are dominated by equids (MNI = 27). The lot is completed by three cattle, two pigs and a dog (Table 1). It is noteworthy that the radiocarbon datings of BO30 and EQ35 are identical (Table 3 and S4 Fig).

The assemblage of sacrifices of this phase consists for the most part of complete skeletons (18 equids and one pig) followed by partially articulated cases (two equids, one cattle and one pig) distributed throughout the courtyard, particularly in the SE quadrant (Table 1). Certain are missing specific anatomical parts. EQ36, for example, lacks its right hind limb while SUS57 is missing its left forelimb, has a fresh bone fracture affecting the mid-diaphysis of its left tibia and bears disarticulation marks on its calcaneus. Other partially articulated animals are missing many of their bones. For example, very few coccygeal and caudal vertebrae were recovered, undoubtedly due to intrusive scavengers, destruction by fire or anthropogenic actions.

The equids and cattle of this first phase are adults, mostly males, and reveal no predominance of laterality, that is, no tendency of being placed on one side or another (Table 1). The equids, notably EQ1-EQ2 and EQ35-EQ36 (Fig 2), are deposited in pairs presumably based on similarities of age and morphometrics. There is likewise a group of four (EQ3-EQ6/EQ4-EQ7) in the SW quadrant arranged in two pairs following the identical orientations. Otherwise EQ16 and EQ38 suffered fractures and the collapse of the upper maxilla caused by a blow yielding a characteristic fracture potentially linked to slaughter (S6 Fig).

Most of the almost complete skeletons reveal no direct evidence of anthropogenic evisceration. Some do bear traces possibly evisceration bucco-pharyngeal or thoracic. This is based on the absence of hyoid bones among 10 equids. Only in one individual cut marks were identified on the ventral side of the ribs indicating possibly abdominal emptying, although it is true that this practice hardly leaves any traces on the bones.

Many of the bones of this phase exhibit different degrees of thermoalteration (Table 1). According to the Munsell chart, the brownish hues (dark brown, reddish-brown, reddish-yellow) align with temperatures between 225 and 350ºC attained by open fires. Some of these features were identified among a relatively thick stratum (15 cm) of wood and barley spikes, which could have been ignited yielding incandescent embers either just before or once the corpses were put in place. The shape of the courtyard delimited by walls containing a mass of bodies packed together could have also generated a closed oven-like environment leading to the toasting effect visible on bone surfaces. This also explains the dehydration of the parts bearing less muscular mass such as the heads and the bone groups of the hooves, precisely those most affected by heat. The thermoalterations in these cases do not occupy the entire surface of the bone. The exceptions are EQ1 and EQ2, which were completely dehydrated resulting in light brown and toasted surfaces (S7 Fig). The non-uniform coloration and the extent of the thermal alterations suggest incomplete burning and protection by the animal’s skin.

Subsequently, the courtyard during Phase 1 accumulated sediments that began to partially cover some of the carcasses. This is deduced from the degree of articulation and the movement of certain labile and persistent joints (e.g., parts of EQ35) and the absence of complete limbs (BO30 and SUS57), which were probably removed during their decomposition in the open air prior to being covered [108]. However, the fractures and dislocations in the skulls of EQ7 and EQ45 require another interpretation as their corpses were apparently subjected to anthropogenic displacements once skeletonized (S8 Fig).

There is also evidence pointing to a dispersal of bones by canids based on bite marks and the dislocation of humerus BO30 found a meters away from its original deposition in the SW quadrant. The bites, according to their shape and size, were produced by dogs. Furthermore, one horse (EQ6) reveals traces along the outer surface of its ribs in the form of shallow scores and striae which are compatible with large scavenging birds such as vultures.

It is noteworthy that certain skeletons (BO28 and BO55) of Phase 1 display striations that do not stem from scavenging but from weathering. These marks follow a different distribution, limited to surfaces exposed to atmospheric agents. Their features and location are compatible with WS 2/WS 3 stages of weathering [93, 94], characterized by the flaking of their surface and at times by the exposure of the fibrous inner tissue. Evidence of these advanced stages of weathering suggests that the bones were exposed for about 2–8 years before being covered by earth (S9A and S9B Fig). In any case, the skeletal overlaps, degrees of articulation and taphonomic marks suggest that the depositions of Phase 1 were not synchronous but took place over a period of several years.

A second type of bone assemblage of Phase 1, collected from the SE quadrant, consists, according to the MNI analysis, of 400 isolated non-fractured bones of nine equids and one dog (EQ101-EQ109 and CA34, Table 1). Certain of those of equids reveal partially articulated anatomical parts such as the distal ends of limbs or segments of the spine and ribs. This potentially indicates that these nine equids were deposited whole.

The unconnected skulls, mandibles and pelvises place the age of four individuals between 5 and 9 years. The information as to sex based on the canines and the morphology of the pelvises suggest they are males. Certain exhibit carnivore (Fig 3) and scavenging birds marks (Fig 4). A small number of appendicular elements (NISP = 16) point to actions compatible with canids: dragging of the diaphysis, punctures and gnawing of the articular ends leading to the partial destruction of the epiphyses. Likewise, a limited number of ribs (NISP = 8) bearing shallow draggings and striae fit the pattern of marks produced by large scavenging birds such as vultures.

The most abundant anatomical elements, with a relative frequency surpassing 70%, correspond to skulls, radiuses, pelvises and femurs. The lumbar and caudal vertebrae, ribs, carpals and sesamoids, by contrast, are under-represented (9–15%). Their dispersal is compatible with actions of canids and scavenging birds [85, 87, 109]. Moreover, certain cut marks identified on the ventral side of two ribs suggest an intentional emptying the abdomen.

Weathering marks such as cracking of the cortex due to prolonged exposure to the sun are visible on metapodials, phalanges and carpal-tarsal bones. Given the scarce muscular mass covering them, they are more easily exposed to the elements once the skeletonization process began. Due to the degree of alteration, they are ascribed to the WS 1 stage of weathering, that is, the fine cortical cracking indicates exposure from between 6 months to 2.5 years [94] (S9C Fig). Other remains bearing superficial chromatic contrasts, notably bleached areas, are indicative of differential sun exposure [110].

The microstratigraphic analysis of the layer of Phase 1 indicates that the animal sacrifices were laid, depending on the preservation of the preceding Phase 0 level, either on the initial sandy gravel or on the subsequent beaten raw earth floor (**Fig 7 in** S1 Appendix). The sacrifices of Phase 1 reveal numerous anthropogenic residues related to the human activities: charcoal, fat-derived charging, charred organic matter, and mainly, bones associated with abundant authigenic phosphate nodules evidencing the presence of animal flesh and *in situ* decomposition (S1 Appendix). In this context, bones bearing serrated edges produced by cyanobacteria after sedimentation and burial suggest the presence of attached organic tissues and flesh. Sedimentary features present within the bone pores are indicative of the environmental conditions during and after the deposition of the animals. In this sense, silt to fine sand-sized aggregates of humid organic matter present within the spongy elements of the bones can be associated with humid remains of organic tissues. Furthermore, laminated silt coatings and post-depositional features of Fe/Mn staining indicative of waterlogging are abundant within the bone pores and surfaces. Similarly, Fe/Mn features are present in the sedimentary matrix containing the bone assemblages. Fe/Mn nodules indicate conditions of long-term postdepositional waterlogging and water saturation after the carcasses were deposited and sealed by the sediments [111]. Thus, the humic acids serving for precipitation and Fe/Mn reduction and mobilization processes would have affected both the bones and in their sedimentary matrix. Water infiltration and percolation towards the lower sediments led to Fe/Mn features forming also in the beaten earth floor of Phase 0. Both the context of the mass sacrifices and the raw earth floor reveal bog iron hypocoatings composed of optically isotropic Fe oxides with radial acicular goethite crystals and ferrihydrite nodules [100]. These features which form in postdepositional waterlogging oxidizing settings indicate contrasting microenvironmental conditions with changes in the redox state. Moreover, polyconcave pores formed in this layer due to water saturated conditions. Finally, a few weathered herbivore coprolites were identified consisting of anatomically connected phytoliths and organic matter.

#### Phase 2

The timeframe of Phase 2, consistent with the dating of Phase 1, is based on the radiocarbon datings of two equids EQ13 and EQ17 (Table 3). This second phase comprises ten almost complete and two partial adult male equids. EQ10, EQ13, EQ17, EQ22/33, EQ23, EQ26/28 and EQ27/31 deposited overlapping each other towards the center of the courtyard in front of the staircase on the slate slab corridor. This occurred presumably after removal of remains of the earlier Phase 1 with the exception of three equids in the SW quadrant (EQ18, EQ19 and EQ61) (Fig 2). Although only five (EQ11, EQ13, EQ17, EQ22/33 and EQ23) bear clear signs of thermoalteration (Table 1), most are on a thick layer of burnt barley ears. The crossed necks of EQ11 and EQ12 (Fig 2), at the foot of the staircase, symbolically blocked off access to the first floor of the building.

A few of the equids of Phase 2 also reveal signs of dislocation of their persistent joints. The presence of dislocations in the skull of EQ19, the segmentation of the body of EQ27/31 into two halves and the displacement of the axial units of EQ18 and EQ61 suggest their carcasses were moved during a stage of their skeletonization (Fig 2).

Although there is evidence of possibly postmortem evisceration of the thorax of EQ17, EQ26/28 and EQ27/31 based on the absence of the hyoid, there are no butchery marks indicative of consumption. These remains also bear no traces of carnivores or necrophagous birds. However, certain (EQ17, EQ27/31) reveal striations due to exposure to atmospheric agents corresponding to the WS 2/WS 3 weathering stages [93, 94] suggesting that they were exposed to the open air for between 2 and 8 years (S9 Fig).

#### Phase 3

The final phase identified in the courtyard consists of an intentional accumulation (ca. 15 cm thick) of seven sacrificed animals (Table 1). The five distributed throughout the NW quadrant next to the staircase correspond to two partial equids (EQ14 and EQ15) two cattle (BO62 and BO29) with articulated segments, one partial and the second almost complete (BO29), and a partial sow (SUS41). The assemblage also consisted of the remains partially articulated of an almost complete sow (SUS59) and a largely complete non-articulated calf (BO56) deposited in the SW quadrant near the southern wall. The range of the radiocarbon dating of BO56 places this sacrifice a few years after that of equids EQ13 and EQ17 of Phase 2 (Table 3 and S4 Fig).

**Fig 5:**
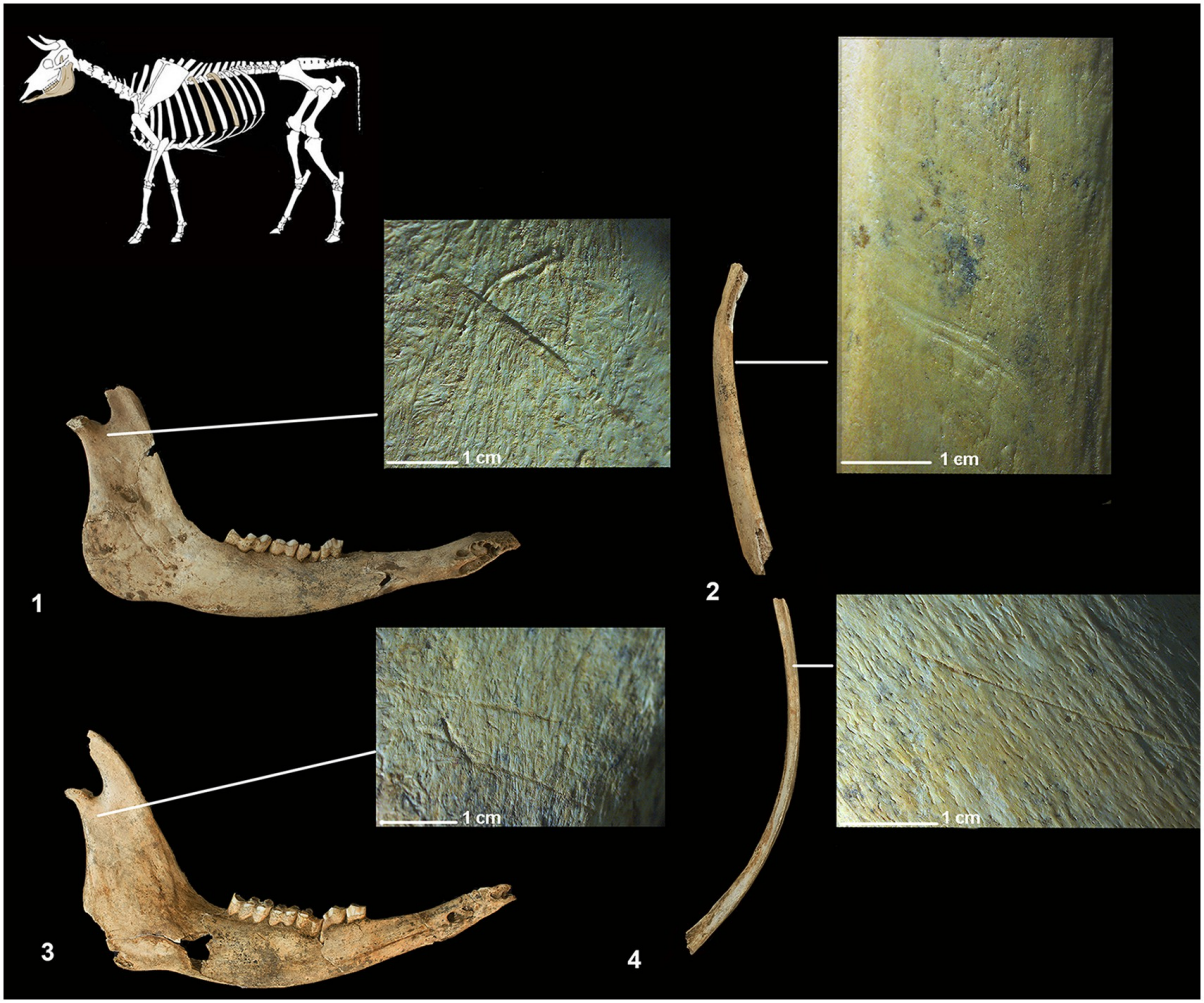
Bones of BO29 bearing cut marks evidencing disarticulation and defleshing during Phase 3. 1–3) disarticulation cuts on the neck of the condylar process observed on both the lateral and medial surfaces of the mandible. 3–4) the traces of defleshing on the inner/lateral surface and caudal border of the ribs.

**Fig 6:**
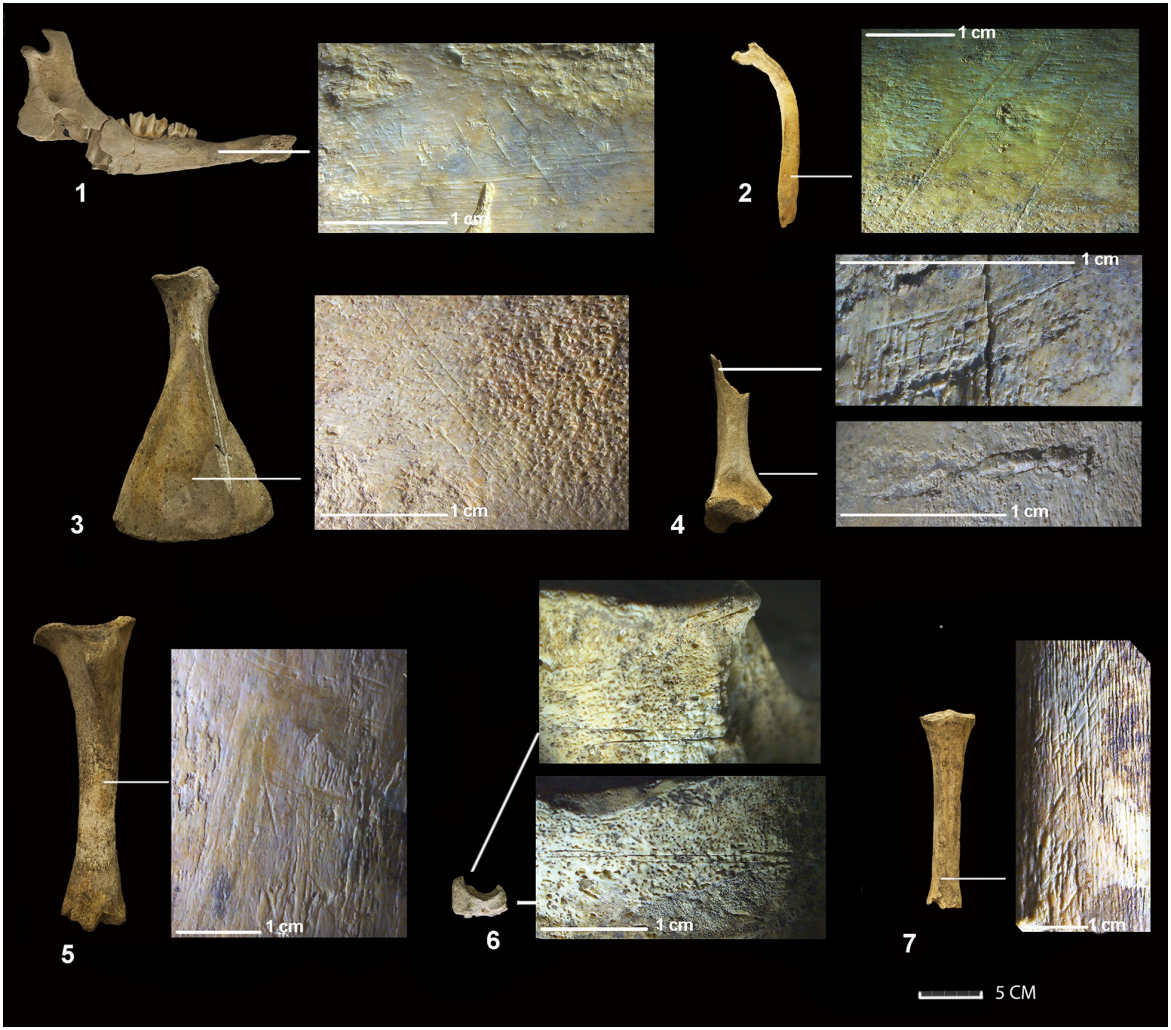
Bones of BO56 bearing cut marks evidencing disarticulation and defleshing during Phase 3. The defleshing marks are visible on a mandible (A), rib (B), scapula (C), humerus (D), tibia (E) and metatarsus (G). The disarticulation cuts are located on the medial and lateral side of centrotarsale bone (F).

**Fig 7:**
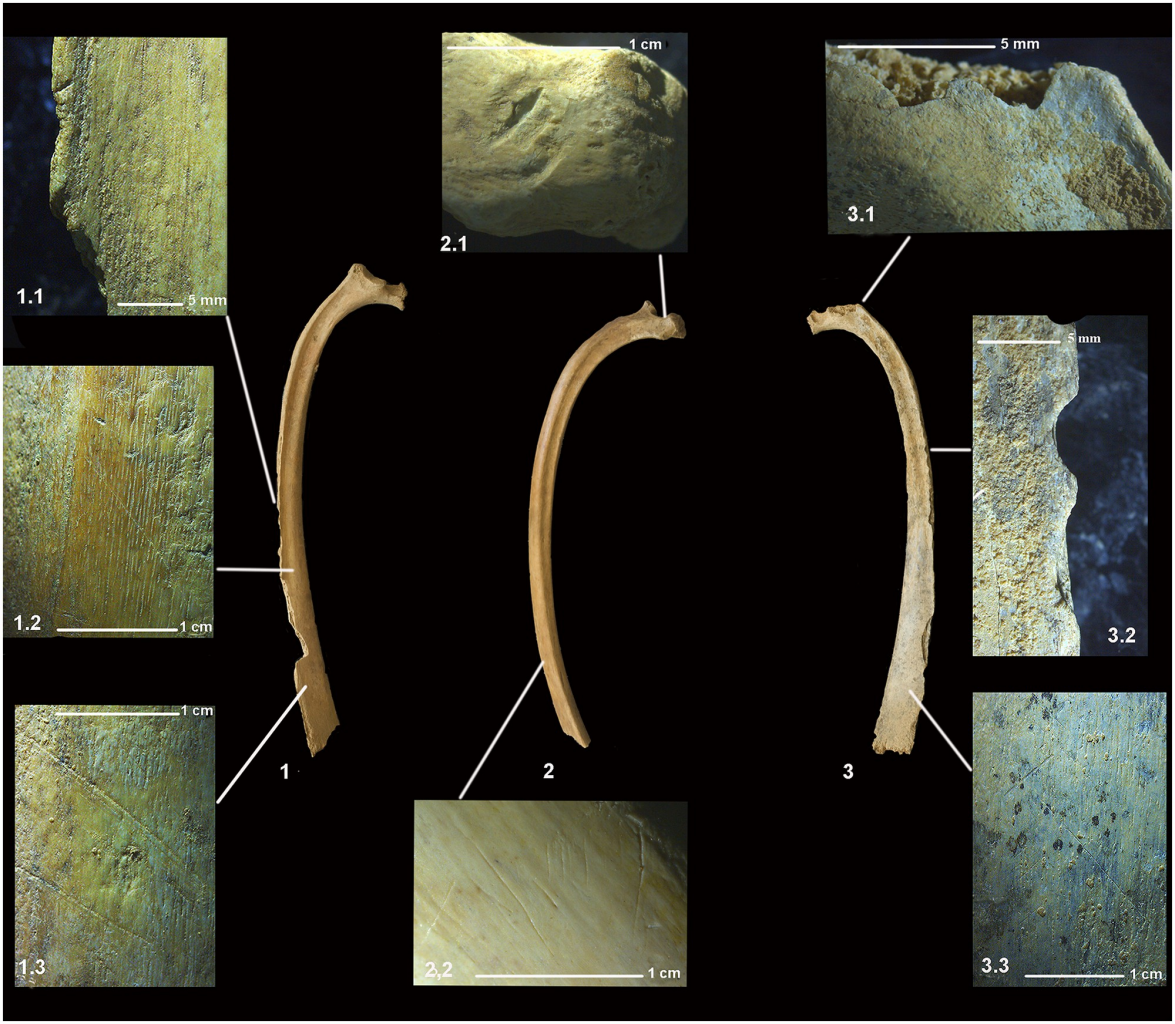
Bones of BO56 bearing cut marks and human bits evidencing defleshing and consumption during Phase 3. The set of ribs (1-2-3) reveal both defleshing (1.2–1.3–2.2–3.3) and human bites on the articular surfaces and on the cranial and caudal edges of the ribs (1.1–2.1–3.1–3.2).

The equids identified in this final phase are male adults whose remains appear disarticulated and completely burned. They also bear no traces of butchery or consumption (Table 1). The cattle (BO29 and BO62) are two partially articulated adults, the second partially burned. An immature and disconnected male (BO56) presented signs of thermoalteration on its distal femur. BO56 and BO29, in turn, reveal butchery cut marks. The mandibles of BO29 show traces of disarticulation while its ribs bear signs of defleshing (Fig 5). The defleshing marks of BO56 take on the form of transverse draggings along the diaphysis of the humerus, radius, femur, tibia and metapodial (Fig 6). In addition to the traces of defleshing, the ribs preserve signs of human bites in the form of jagged outlines on their articular surfaces, on the anterior edges and at the distal ends (Fig 7).

The pigs are all females between two (SUS59) and three (SUS41) years of age (Table 1). Sow SUS59 in a partially articulated state appears to have been deposited whole. SUS41, in turn, is only represented by its skull and a thermoaltered anterior appendicular left limb.

The degree of weathering of all the skeletal remains of Phase 3 corresponds to the WS 0 (i.e., an unweathered stage). The microstratigraphic analyses of the sediments of this layer indicate massive mineralogical microstructures dominated by silt to sand-sized quartz bearing pseudomorphic moldic voids stemming from the decay of organic matter. This characterization falls in line with the dissolution of the site’s construction materials, specifically the mudbricks used that served to raise the structures (**Fig 8 in** S1 Appendix).

## 5. Discussion

The 52 animals showed in the courtyard of Casas del Turuñuelo evidence a repeated use of this space for animal sacrifice. These animals, mainly horses but also cattle, pigs and a dog, formed part of a succession of ritual practices or events carried out in different phases and motivated by unknown reasons. Also unclear is the number and role of the humans coordinating the rites.

A research gap exists on the subject of mass sacrifice, specifically on the choice of animal, species and sex, between the archaeological record and classical written sources which at times refer to mass public sacrifices of oxen known as hecatombs. The existence of this gap therefore renders it difficult to apply a precise term, apart from that of “Mass Sacrifice,” to describe the rites practiced at Casas del Turuñuelo.

The results of the taphonomic and microstratigraphic analyses indicate that this site saw various phases, a fact that reduces the number sacrifices by phase. Even so, the volume of sacrifices of Phases 1 and 2 is extraordinary for European Iron Age contexts, especially considering that Phase 1 consisted of 27 equids, three cattle, two pigs and a dog, more than double that of Phase 2 (12 equids). The fact that the number in the final Phase 3 drops dramatically (two equids, three cattle and one sow) and that their characteristics differ suggests changes of rites with respect to the two earlier phases.

In any case, the evidence allows defining a succession of rituals aligned with specific choices of animal species, ages and sex, and how they were arranged. The assemblage reveals a predominance of equids (MNI = 41), that is, horses, a donkey and possibly a few hybrids, followed by cattle (MNI = 6), pigs (MNI = 4) and a single dog. Adult males are predominant among the equids and cattle. The pigs, in turn, correspond to adult and subadult females reflecting a specific choice for a different sacrificial act. Age profiles reveal as predominance of adults and the absence of young and subadults (except for pigs and a calf). These ages in fact eliminate the hypothesis of a natural or epidemic death. The evidence of the gestures and sequences related to the deposition of certain equids in fact point to “sacrificial stagings” accompanied by the burning of barley offerings and sumptuary goods. This staging is also evidenced by the arrangement in pairs of male equines of similar height and age. EQ11 and EQ12, for example, were deliberately placed at the foot of the stairs, facing each other and with intertwining necks (Fig 2). Moreover, they are preceded by other animals literally sealing off the path along a slate corridor leading from the eastern gate to the foot of the stairs accessing the second story of the adjacent building. Similarly, EQ1 and EQ2 are opposite each other to the southwest of the stairs. Finally, EQ35 and EQ36 were placed back-to-back near the eastern door (Fig 2, quadrant SE) as are two other pairs (EQ3 and EQ6; EQ4 and EQ7) in the SW of the courtyard.

This arrangement of equid pairs bearing similar characteristics raises the possibility that they were cart animals sacrificed simultaneously. The idea is bolstered by their pathologies indicative of having served for riding and possibly to pull carts. The discovery of metallic bits *in situ* coupled with premolar wear and erosion of the gaps among more than half of the equids prove they were handled and controlled [96–98] and thus served for work. Osteoarthrosis of the proximal interphalangeal joints are pathologies commonly associated with saddle animals and agricultural work [112, 113]. Others bear vertebral exostoses resulting from spondylosis [114], lesions that can stem from saddles [115] although are also common among non-riding animals. Current clinical studies suggest that there is no clear relationship between a riding technique and spondylosis, nor between the degree of effort and the presence of bone alterations [116].

The ritual use of fire is also clearly evidenced throughout the different phases in the form of a thick layer (15 cm) of charred barley ears, vegetable fiber mats and wood fragments under the remains of 18 equids, one cattle and two pigs. The total charring of the bones of EQ11 and EQ12, for example, point to the intentional burning of both cereals and animals. Moreover, the animals during Phase 2 were accompanied by luxury good from the eastern Mediterranean, items that reinforce the importance of animal sacrifices. The goods comprise three Punic vitreous paste ointment jars, four Macedonian bowls, a fragment of a sculpture of Athenian Pentelic marble [47] and a set of five sheep astragali with signs of abrasion along their lateral and medial facets. The deposition of these elements associated with games of chance and divination is in fact common to Iron Age in cemeteries and sanctuaries [117–119]. A parallel in the Guadiana River Plain is the lot of 300 partly burned, perforated caprine astragali discovered in a hollow in the company of embers and ashes in the courtyard facing the door of the sanctuary of Cancho Roano [12].

It is noteworthy that the events of Phase 3 differ those of the previous phases. Its singularity resides both in the lower number of sacrifices and finds associated with a feast or banquet. This is based on an arrangement of calf bones (BO56) resembling a *bothros* bearing marks of butchery and human consumption. The notion of a banquet is likewise supported by the scattered bones of an adult cattle (BO29) bearing similar traces. Moreover, despite the presence of two equids, only cattle were consumed. This is in line with the type of meat most frequently consumed at other Tartessian domestic sites of the Guadiana Valley [24]. Moreover, there is likewise evidence of the celebration of ritual feasts with the consumption of domestic species (but few or no equids) are also recorded in both funerary and domestic spaces at other Iron Age sites of the Iberian Peninsula notably the sanctuaries of Castrejón de Capote [120], La Algaida [121], Carambolo Bajo [122], Alhonoz [123], Garvão [124], Amarejo [125] and the settlements of Mas de Castellar de Pontós [126], Alorda Park [127, 128], Puig de Alcoy [129] and Bastida de las Alcusses [130].

The deposition of unconsumed horses in the Turuñuelo courtyard is unique and has few parallels. A first aspect to highlight is the quantity and the deliberate choice of animals between 5 and 7 years, their age of maximum value as riding or draft animals. It is also noteworthy that equids at other Tartessian sites are not only scarce but rarely bear signs of consumption. Hence, their sacrifice in the context of a unique monumental building is also remarkable as these domestic species possessed great added value for their role in work and as an asset of prestige. However, the equids in other non-funerary Iron Age ritual sacrifices in Iberia do not precisely replicate the patterns of Casas del Turuñuelo. The site with the most similarities is the aforementioned Cancho Roano with a minimum of 13 unconsumed horses and donkeys, most disarticulated, whose heads were placed far from their post-cranial remains.

Special depositions comprising complete or partial equids are known elsewhere in the Iberian Peninsula in production areas and in isolated pits of settlements. However, their number never approaches that of the courtyard of Casas del Turuñuelo. Some of the most outstanding examples from the Early Iron Age are the horse fetuses unearthed at the Iberian Culture site of Els Vilars (Arbeca, Lleida) [10, 131] and those found in silos at Can Roqueta (Sabadell, Barcelona) [3]. Ritual depositions of equid heads dating from the 6th-5th centuries BCE are also recorded in the settlement of Puig d’Alcoi (Alcoi, Alicante) [8], in the production area of La Cervera (Font de la Figuera, Valencia) [8], in the sacred area of Ruaya (Valencia) [8] and in front of the rampart of Tossal de Manises (Alicante) [132]. Other examples from more recent timeframes are a lot of 40 equids deposited over a span of 200 years in an area of silos at Serrat dels Espinyers (Issona, Lleida) [4] and isolated horse burials at La Regenta (Burriana, Castellón) [133]. Horses are likewise clearly associated with human burials and cremations. An example is the complete equid in a silo next to the partial cremation of a woman at Hort d’en Grimau [134], the horse skull recovered next to the skeleton of a male in a silo at Can Roqueta [2] and the partial remains of several horses from the Iberian cemetery of La Pedrera (Vallfogona de Balaguer) [135].

The closest parallels beyond the Iberian Peninsula are the more recent Gallo-Roman sanctuaries of Vertault (Côte-d’Or) [132] and Longueil-Sainte-Marie “L’Ormeon” (Oise) in France [136]. These closed spaces saw a succession of ritual sacrifices of unconsumed horses. The *Oppidum* of Pech Maho [38] from the 3rd century BCE, by contrast, contained various spaces with evidence of the *in situ* slaughter, processing and consumption of 35 equids (including 3 donkeys). Moreover, this site reveals a preference for young adults (5–8 years old) in a framework of collective commensalism marked by a concurrence of warlike and funerary components. Yet the only element these French sites share with Casas del Turuñuelo is the choice of the horse as the main species of the ritual, an aspect that characterizes the communities of the European Iron Age and that is undoubtedly related to the role this animal played as an asset of prestige.

The taphonomic and microstratigraphic analyses carried out at Casas del Turuñuelo thus suggest a succession of depositions of animals, in particular equids. The radiocarbon datings, due to the Hallstatt plateau, are not sufficiently precise to quantify the lapse between the first two phases. However, the combination of the results of analyses stemming from different disciplines suggest the repeated use of the courtyard of the Turuñuelo for sacrifice over a timespan corresponding to end of the 5th century BCE. The evidence as a whole suggests that this building played a key ceremonial role and/or served as a political control center dominating the Tartessian communities of the Guadiana River Plain.

## 6. Conclusion

The observations of the faunal assemblage and the results of the microstratigraphic analysis presented here reveal the complexity of the sequence of layers of Casas de Turuñuelo and the extensive temporal processes involved in their formation. The taphonomic and microstratigraphic analyses identified a series of processes related to the formation of the assemblage as well as human activities before, during and after the mass sacrifice. The evidence garnered at the site therefore suggest guidelines, (except for pigs), for ritual sacrifices ruling the selection of species, mainly adult male equids.

The 52 animals deposited in the courtyard of Casas de Turuñuelo represent a series of episodes of slaughter. Most were male adult equids normally dedicated to domestic tasks. Cattle, sows and one dog were secondary choices. The different episodes reveal that the courtyard, based on both evidence offered by the bones themselves as well as by absolute dates, served for animal mass sacrifice over a span of several years. The first two phases are characterized by complete unconsumed animals, that at times reveal fractures to the head possibly related to their slaughter. They likewise bear evidence of post-mortem evisceration. Traces of scavengers also explain the dispersion of the bones, especially those of Phase 1 in the SE quadrant, also explain the dispersion of bones. Phase 3, by contrast, is characterized by cattle bones bearing clear signs of processing for human consumption.

The different episodes were certainly linked to a variety of ritual acts such as the display over years of sacrifices, unique actions with few parallels. Moreover, large quantities of burned barley ears and certain animals from Phases 1 and 2 suggest that fire played a key role in the rituals. However, the rites changed in Phase 3. In spite of the persistence of the sacrifice of certain types of animals in this last phase, others reveal clear evidence that they were consumed by humans, potentially in the framework of a meal or banquet prior to their deposition in the courtyard as tokens of the event.

## Supporting information

S1 Fig Artifacts recovered in the courtyard. A: three Punic ointment jars made of vitreous paste. B: four bowls of Macedonian origin. C: a fragment of a sculpture carved in Pentelic marble. D: five sheep astargali bones (4 from the left foot and 1 from the right), with the lateral and medial facets modified by abrasion. a) plantar side. b) dorsal side. c) lateral and medial facets.
(TIFF)

S2 Fig Physical trauma to the jaw and teeth caused by metal bits to the horses. A: wear to the upper and lower PM2 of EQ9, B: EQ1 bit, C: bone spurs on the diastema of female EQ20.
(TIFF)

S3 Fig Vertebral body abnormalities to the horses. A: thoracic vertebrae of EQ45, T13-T18, lateral view, osteophytes affecting the anterior and posterior joints of the ventral area. B: thoracic vertebrae of SE quadrant, T5-T6, lateral view, osteophytes affecting the ventral area and intertransverse space. C: thoracic vertebrae of the SE quadrant, T15-T16, lateral view, vertebral ankylose osteophytosis.
(TIFF)

S4 Fig Calibrated ranges for the Turuñuelo ^14^C dates on samples EQ35 and BO30 of Phase 1, EQ13 and EQ17 of Phase 2 and BO56 of Phase 3. (TIFF)

S5 Fig Schematic profile indicative of the accumulations of bones and sediments (Phases 1–3) in the courtyard. The view is from the eastern entrance of the courtyard toward the monumental western staircase. SP: slate paving.
(TIFF)

S6 Fig View of the maxilla of EQ16 of Phase 1 bearing traces of a fresh blow to the right maxillary sinus possibly indicative of slaughter. (TIF)

S7 Fig Thermoalteration of the bones of EQ2 during Phase 1. The bones of this equid reveal signs of dehydration, cracking and color change along the superficial cortical zone of the radius (A), talus (C) and calcaneus (D). The color changes affected the peripheral cementum of the labial surface of the maxillary dentition (B upper). The lingual surface (B lower) is less affected, probably due to protection offered by soft tissue such as the tongue. The predominance of reddish-brown, reddish-yellow and at times dark brown according to the Munsell chart suggest the bones were exposed to temperatures between 225 and 350º C.
(TIF)

S8 Fig Bone dislocations and displacements of Phase 1. 1. EQ 7: A) incisor bone, B) left maxilla with molars M1-2-3, C) occipital bone, D) axis displacement. 2 EQ35 atlas dislocation, 3–4 EQ35 femur and pelvis dislocation.
(TIF)

S9 Fig Bones of Phases 1 and 2 bearing traces of weathering. A) right hemimandible of EQ17 (Phase 2) revealing flaking and a fibrous texture on its exposed lateral surface (1) but no traces of weathering on its medial surface (2). B) Right tibia of BO55 (Phase 1) revealing flaking of its surface, especially on its exposed medial side (1), as opposed to its caudal surface (2). C) Right metacarpal (Phase 1, SE quadrant) revealing cracking of the cortex due to sun exposure on its exposed distal surface (1) but no traces on its plantar side (2).
(TIF)

S1 Appendix Microstratigraphic analyses of the courtyard. Archaeological sediments.
(PDF)

S1 Table **A)** Anatomical representation and **B)** Withers height stimations.
(XLSX)
