# Supplementary figures and images for: Mass animal sacrifice at casas del Turuñuelo (Guareña, Spain): A unique Tartessian (Iron Age) site in the southwest of the Iberian Peninsula
Source: PLoS One. 2023 Nov 22;18(11):e0293654. doi: 10.1371/journal.pone.0293654 (PMC10664939; doi:10.1371/journal.pone.0293654)

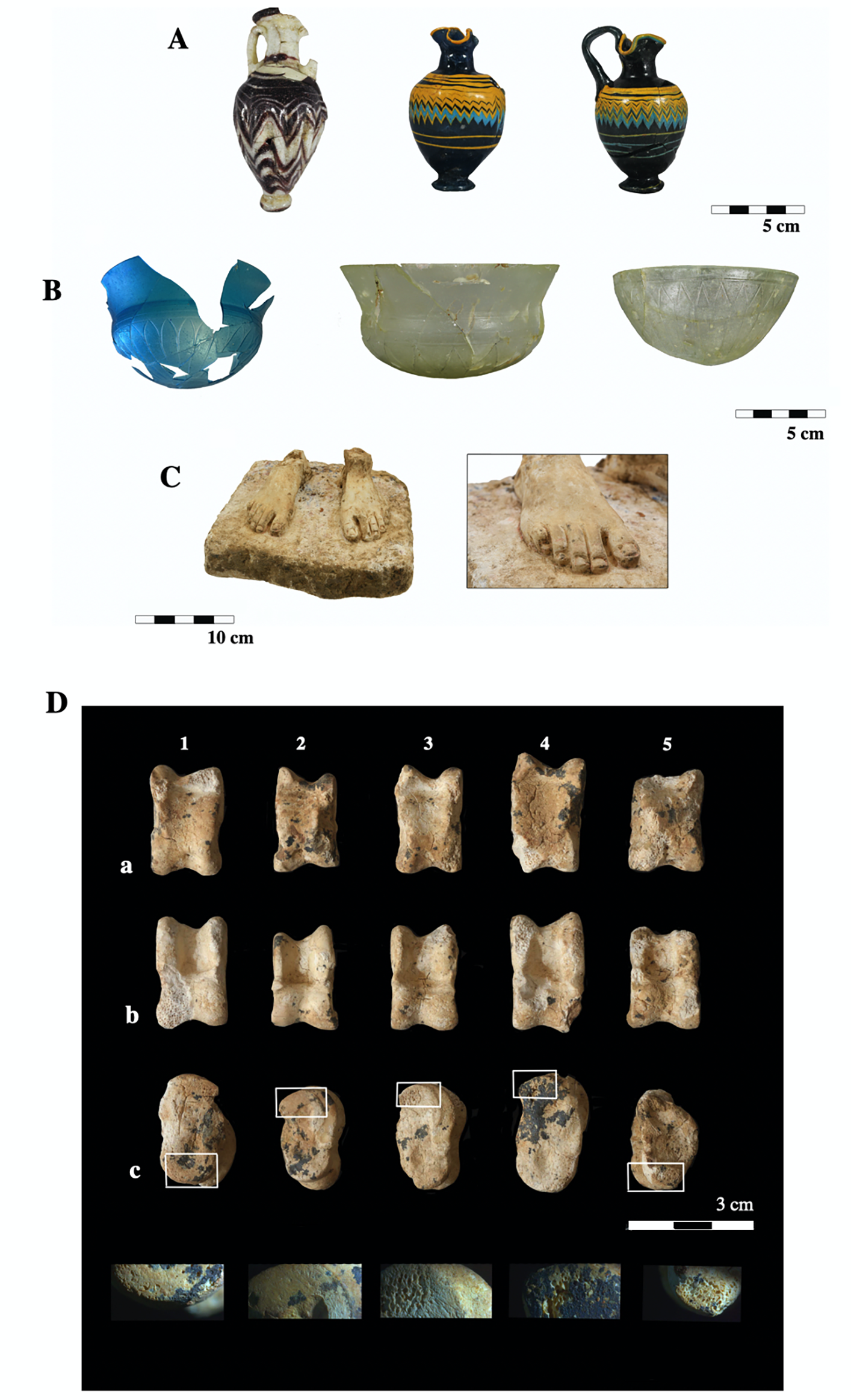

Supplement: S1 Fig — A: three Punic ointment jars made of vitreous paste. B: four bowls of Macedonian origin. C: a fragment of a sculpture carved in Pentelic marble. D: five sheep astargali bones (4 from the left foot and 1 from the right), with the lateral and medial facets modified by abrasion. a) plantar side. b) dorsal side. c) lateral and medial facets. (TIFF) [file pone.0293654.s001.tiff]

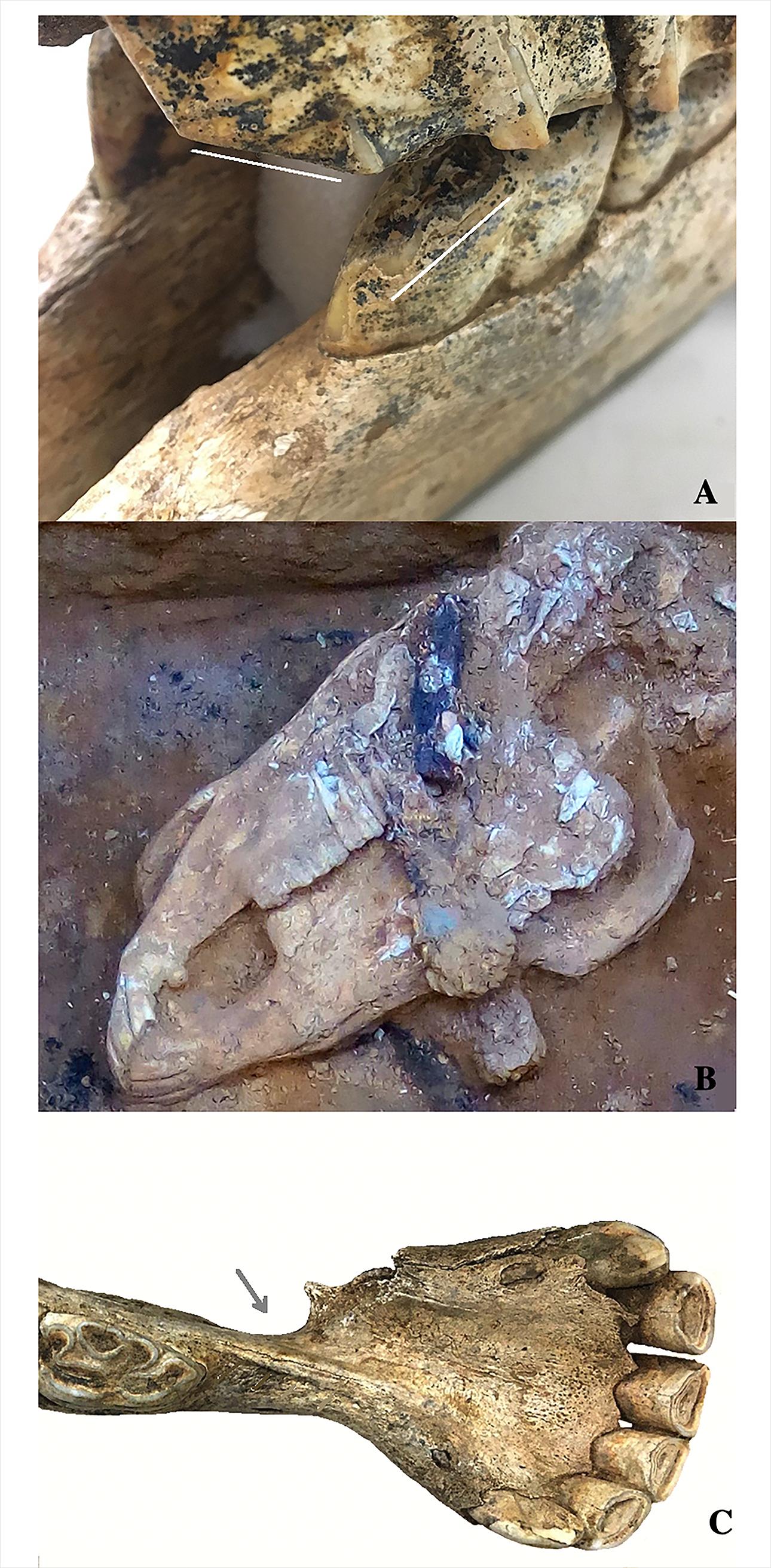

Supplement: S2 Fig — A: wear to the upper and lower PM2 of EQ9, B: EQ1 bit, C: bone spurs on the diastema of female EQ20. (TIFF) [file pone.0293654.s002.tiff]

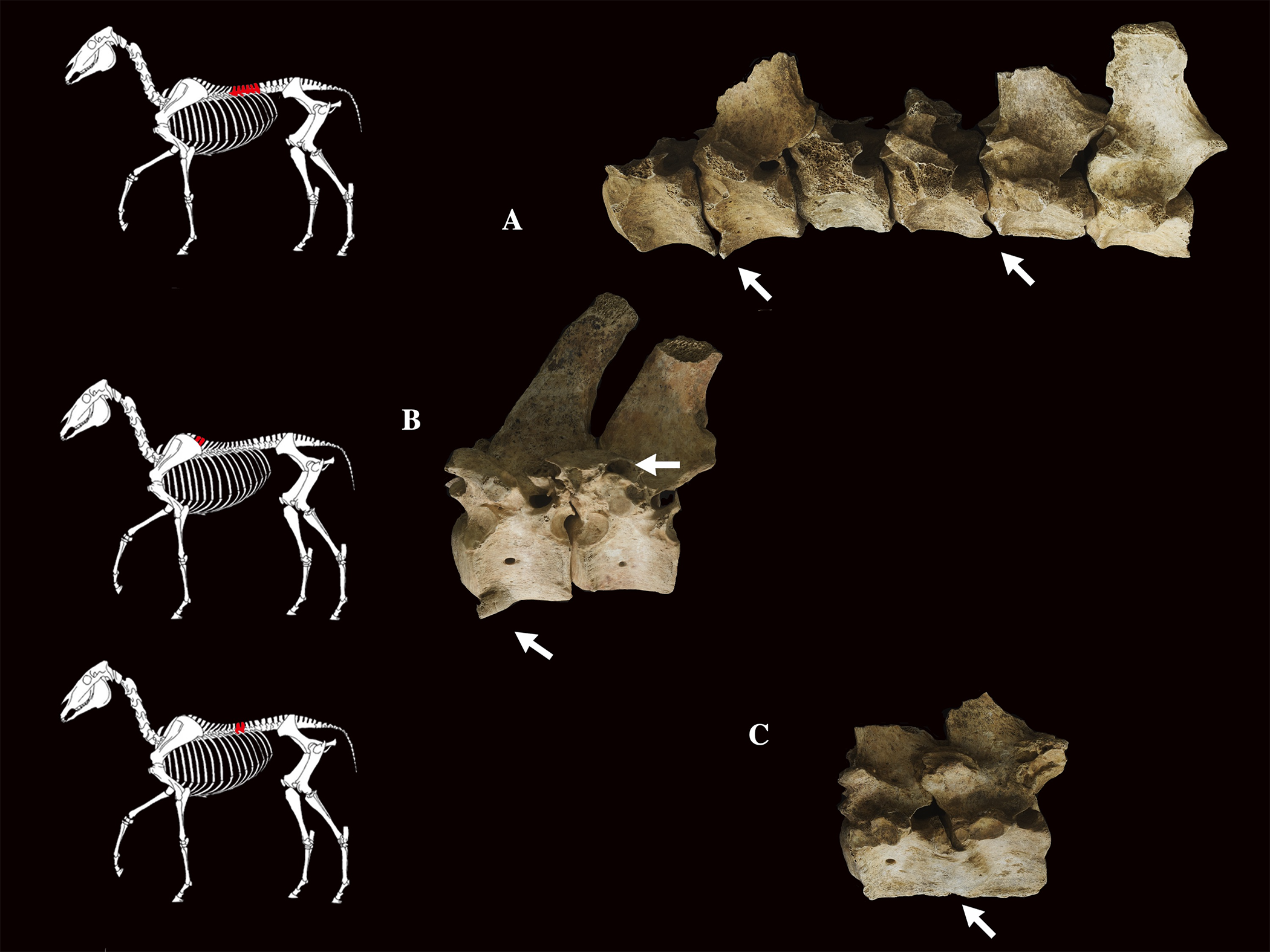

Supplement: S3 Fig — A: thoracic vertebrae of EQ45, T13-T18, lateral view, osteophytes affecting the anterior and posterior joints of the ventral area. B: thoracic vertebrae of SE quadrant, T5-T6, lateral view, osteophytes affecting the ventral area and intertransverse space. C: thoracic vertebrae of the SE quadrant, T15-T16, lateral view, vertebral ankylose osteophytosis. (TIFF) [file pone.0293654.s003.tiff]

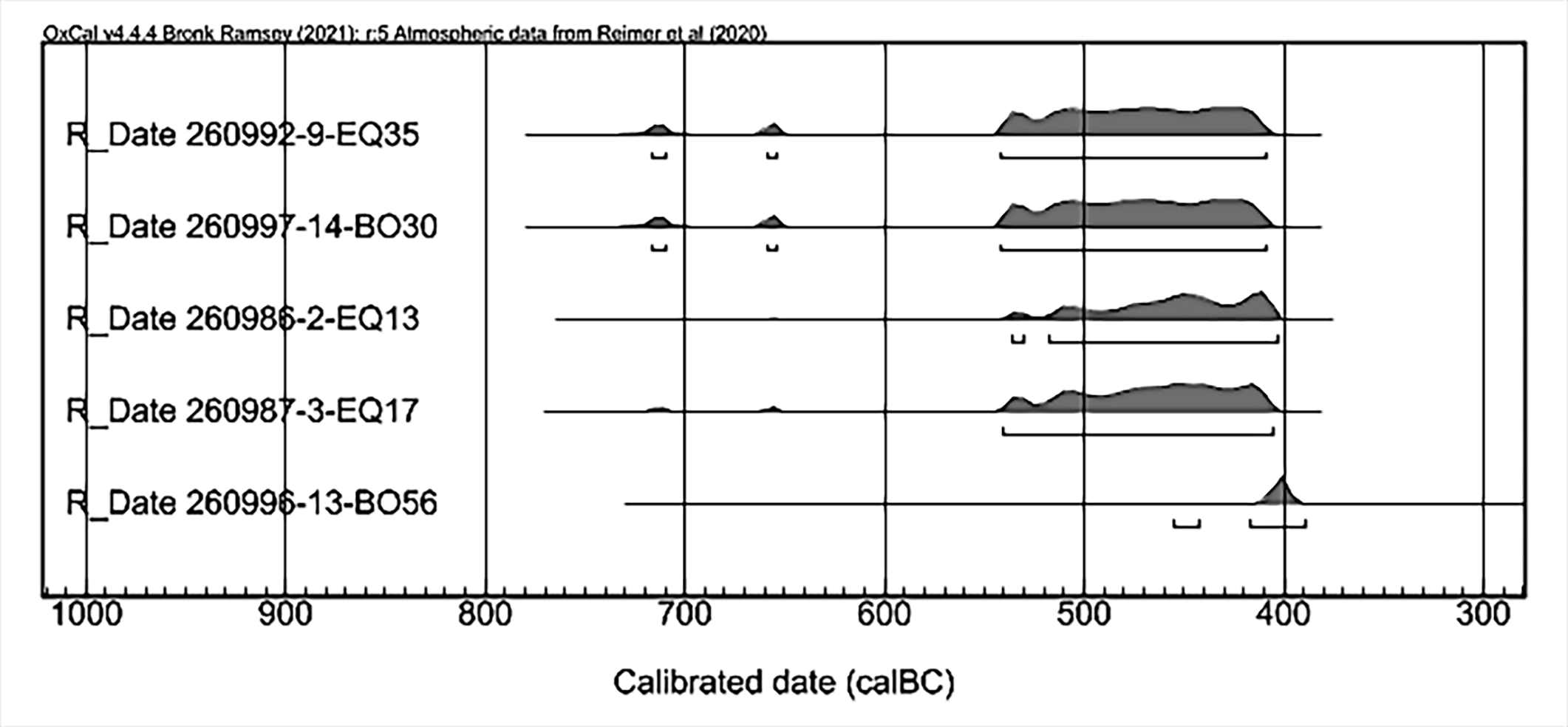

Supplement: S4 Fig — (TIFF) [file pone.0293654.s004.tiff]

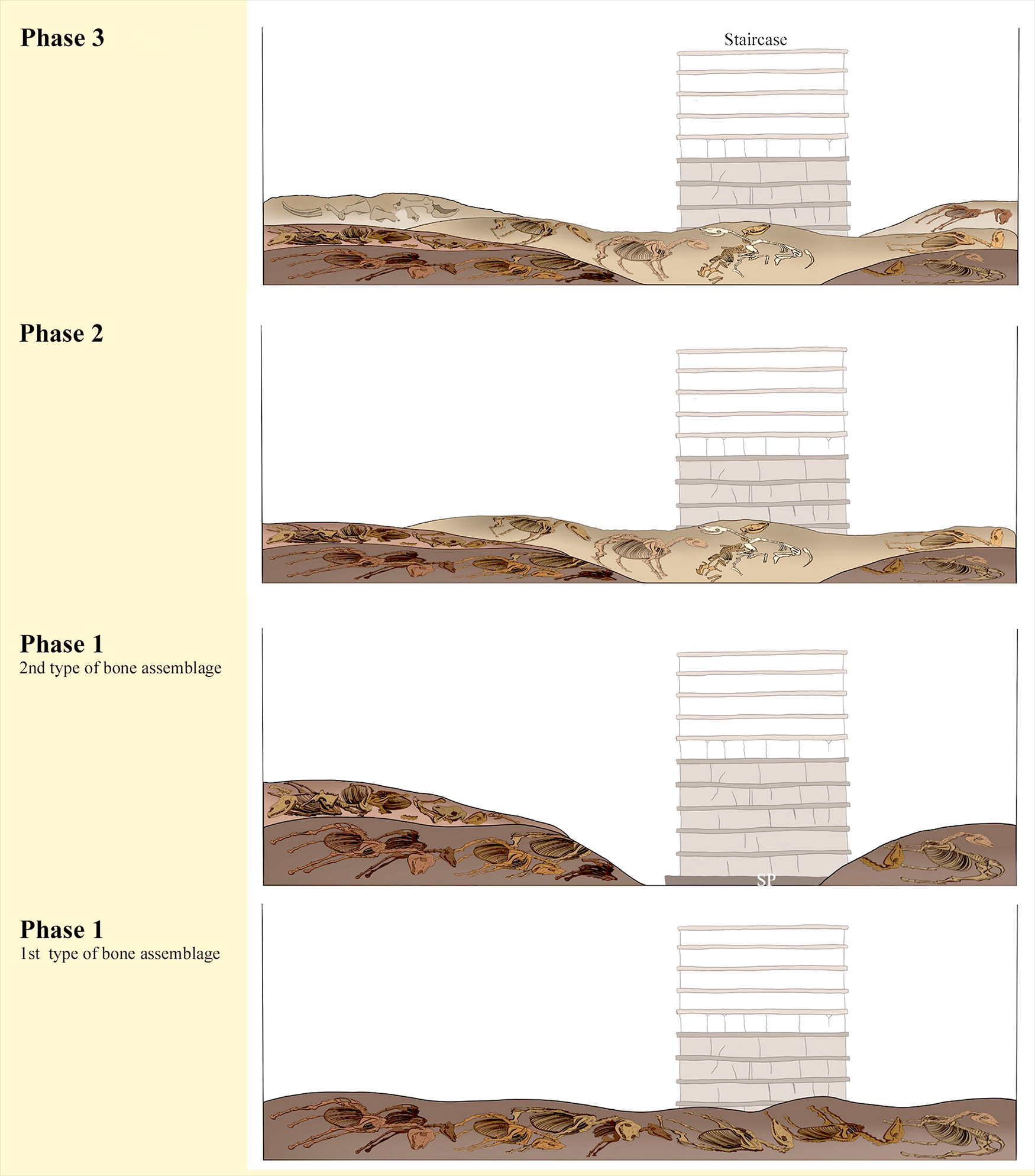

Supplement: S5 Fig — The view is from the eastern entrance of the courtyard toward the monumental western staircase. SP: slate paving. (TIFF) [file pone.0293654.s005.tiff]

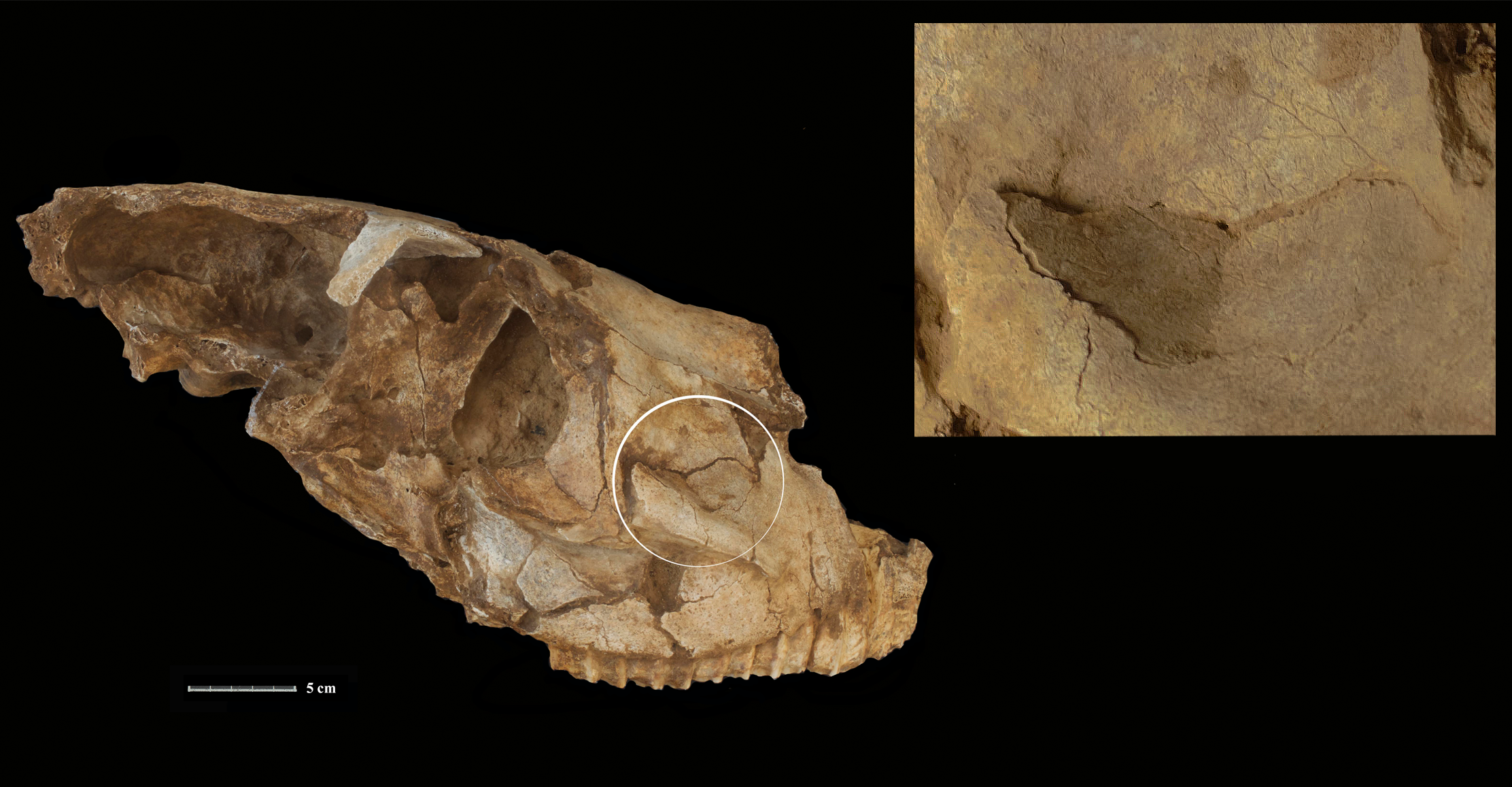

Supplement: S6 Fig — (TIF) [file pone.0293654.s006.tif]

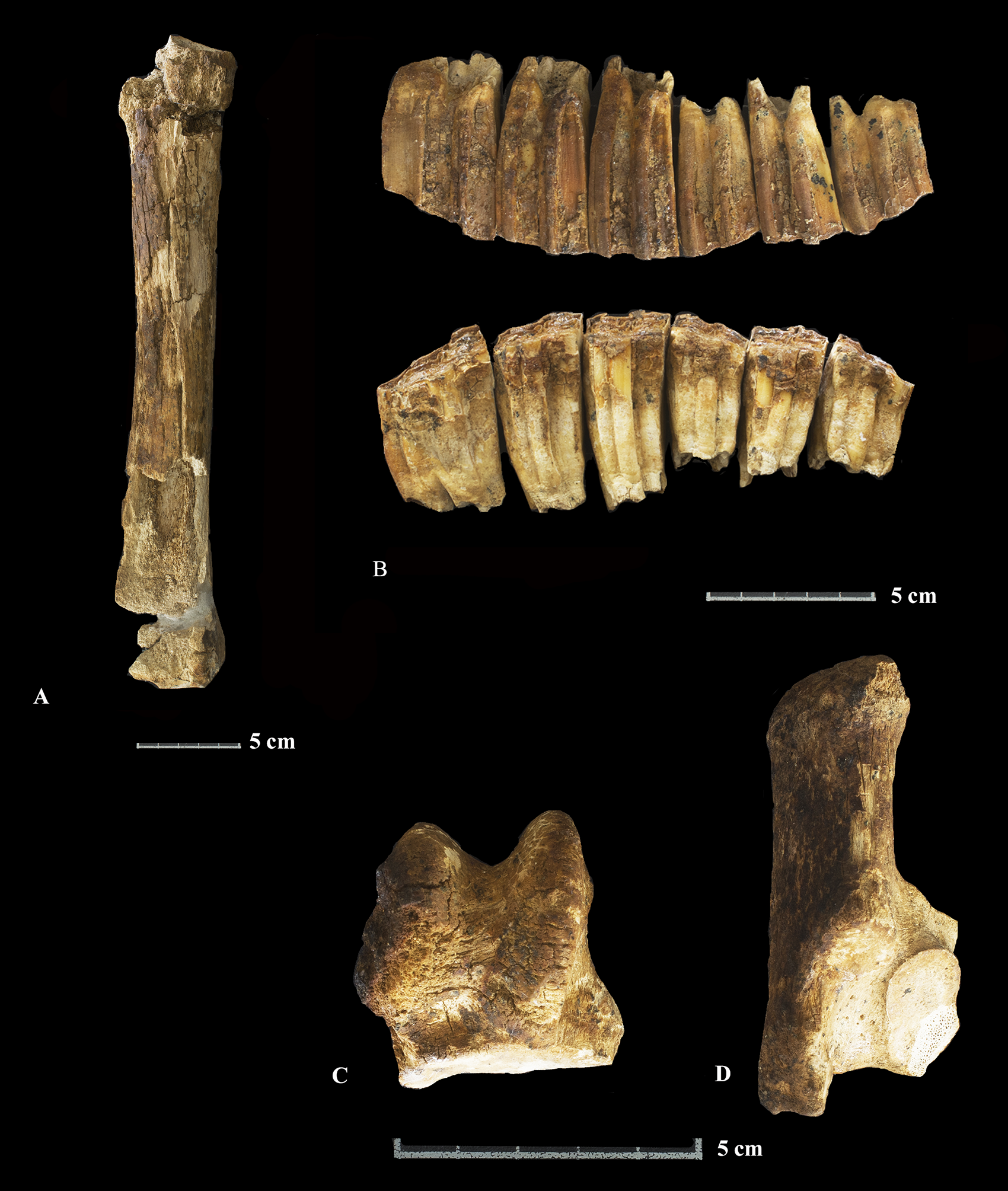

Supplement: S7 Fig — The bones of this equid reveal signs of dehydration, cracking and color change along the superficial cortical zone of the radius (A), talus (C) and calcaneus (D). The color changes affected the peripheral cementum of the labial surface of the maxillary dentition (B upper). The lingual surface (B lower) is less affected, probably due to protection offered by soft tissue such as the tongue. The predominance of reddish-brown, reddish-yellow and at times dark brown according to the Munsell chart suggest the bones were exposed to temperatures between 225 and 350º C. (TIF) [file pone.0293654.s007.tif]

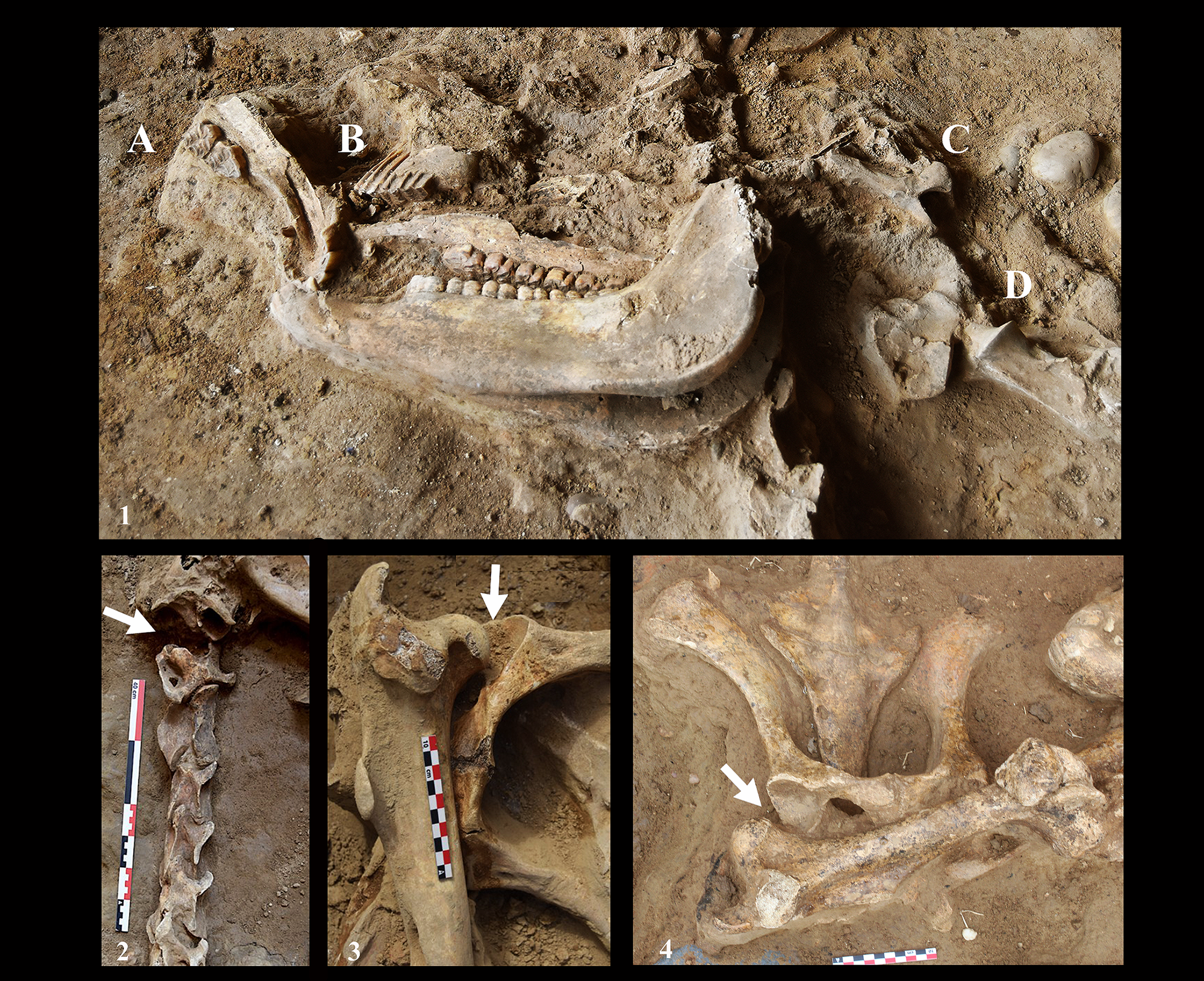

Supplement: S8 Fig — 1. EQ 7: A) incisor bone, B) left maxilla with molars M1-2-3, C) occipital bone, D) axis displacement. 2 EQ35 atlas dislocation, 3–4 EQ35 femur and pelvis dislocation. (TIF) [file pone.0293654.s008.tif]

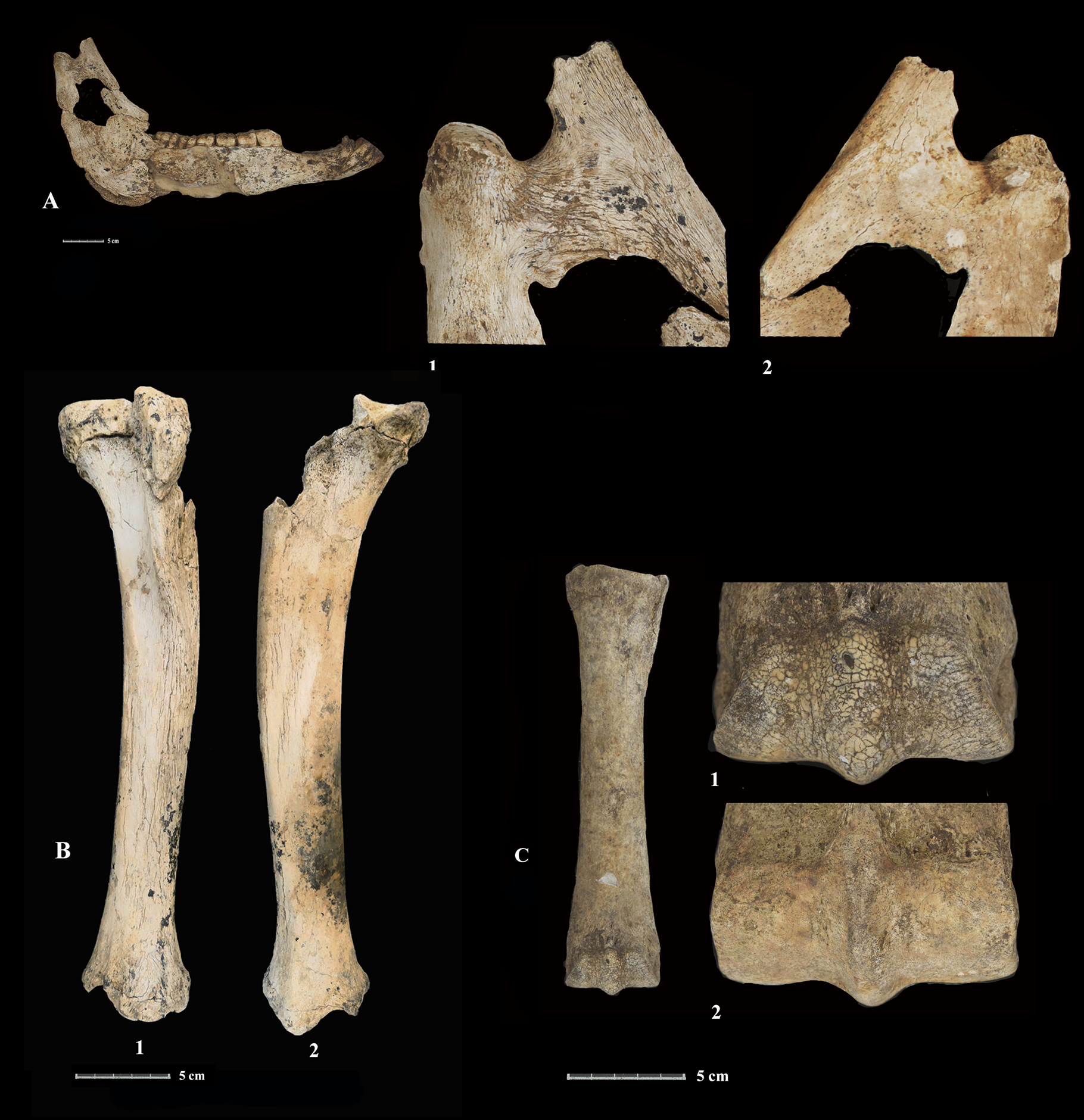

Supplement: S9 Fig — A) right hemimandible of EQ17 (Phase 2) revealing flaking and a fibrous texture on its exposed lateral surface (1) but no traces of weathering on its medial surface (2). B) Right tibia of BO55 (Phase 1) revealing flaking of its surface, especially on its exposed medial side (1), as opposed to its caudal surface (2). C) Right metacarpal (Phase 1, SE quadrant) revealing cracking of the cortex due to sun exposure on its exposed distal surface (1) but no traces on its plantar side (2). (TIF) [file pone.0293654.s009.tif]
