# Supplementary material for: Mass animal sacrifice at casas del Turuñuelo (Guareña, Spain): A unique Tartessian (Iron Age) site in the southwest of the Iberian Peninsula
Source: PLoS One. 2023 Nov 22;18(11):e0293654. doi: 10.1371/journal.pone.0293654 (PMC10664939; doi:10.1371/journal.pone.0293654)
Supplement: S1 Appendix — Archaeological sediments. (PDF) [file pone.0293654.s010.pdf]

# S6. MICROSTRATIGRAPHIC ANALYSIS OF THE COURTYARD ARCHAEOLOGICAL SEDIMENTS

## 1. SAMPLE LOCATION

Five soil micromorphology samples were taken from the mass animal sacrifice context. The sampling strategy was selective. After identifying the different faunistic assemblages, we selected key areas to sample: 1) the sediments on which the assemblages lied; 2) the matrix where the animals were found; and 3) the sediments covering the entire assemblage. From each sample, a thin section was produced.

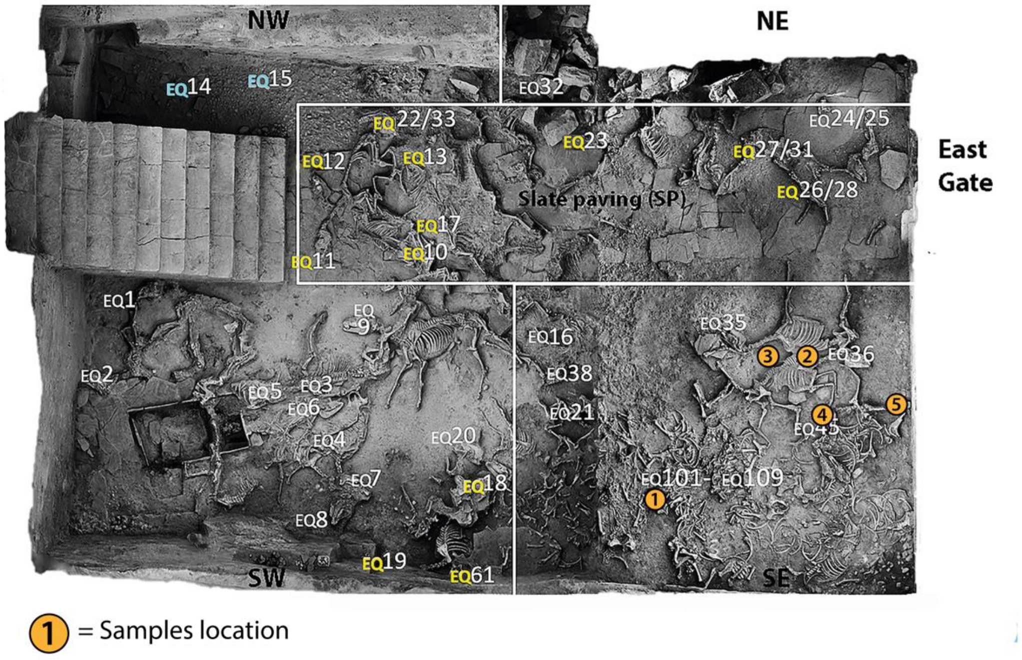


*Figure 1: Plan of the courtyard showing the samples location* (**https://construyendotarteso.com/es/paginasITM/hecatombe-animal**)

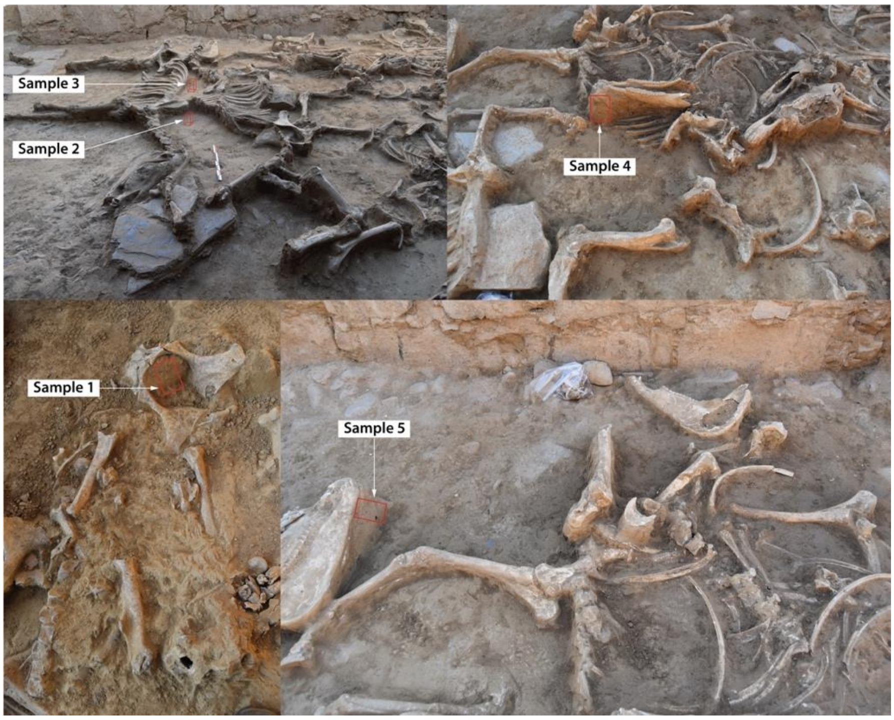


*Figure 2: Detailed contextualization of each of the studied samples*

## 2. MICROFACIES ANALYSIS AND CORRELATION WITH THE PHASES IDENTIFIED

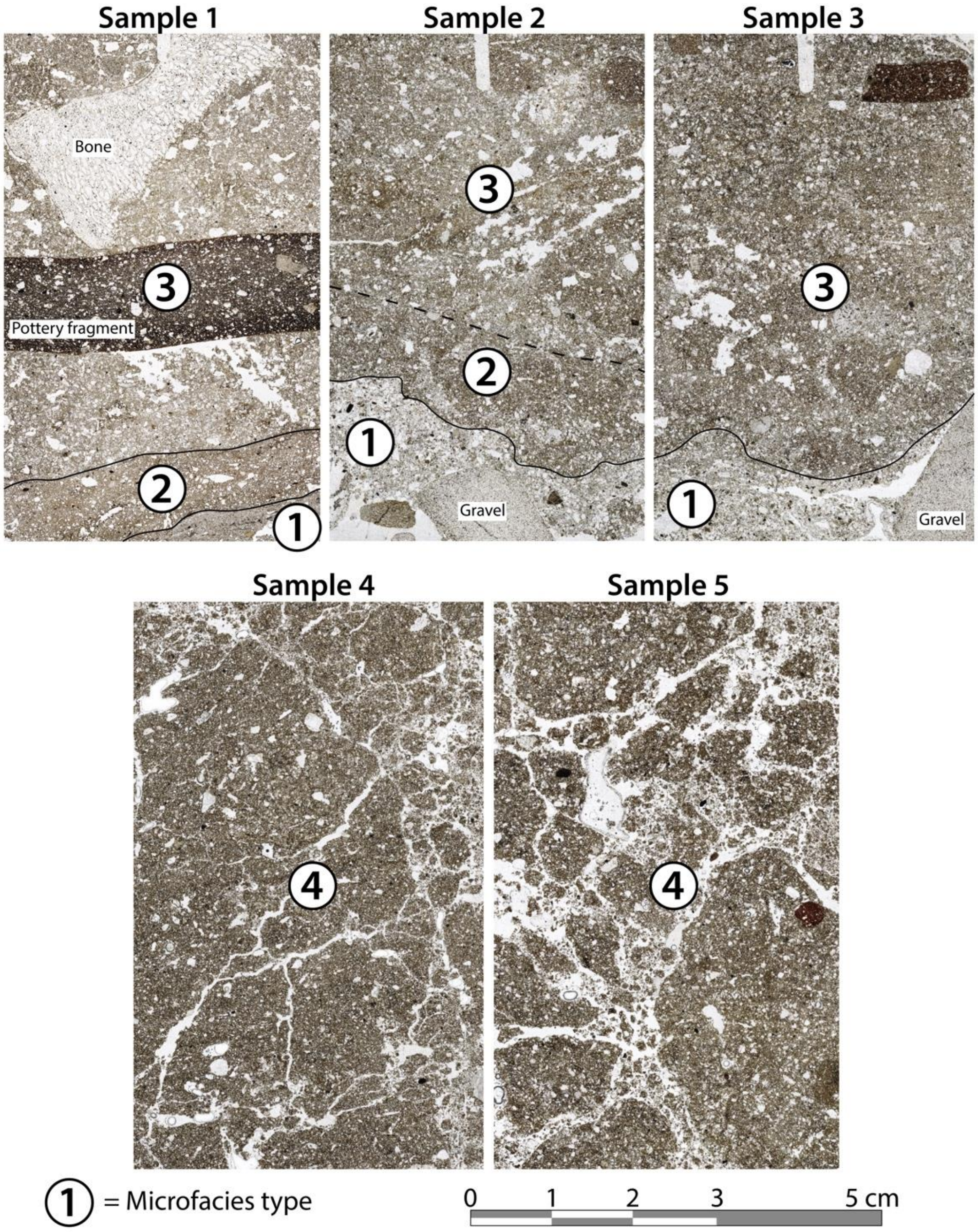


*Figure 3: Microfacies analysis*

Each sample was analyzed under the petrographic microscope by means of archaeological soil micromorphology. Descriptive standards were followed[1–6]. We grouped the sediments observed in thin sections in four different microfacies[7–9], understood as sediments with similar characteristics of lithological composition, geometric association and post-depositional changes. This association allowed us to differentiate distinct events, depositional environments and post-depositional processes involved in the genesis of the mass animal sacrifice context. In this case, the most discriminating factors in defining microfacies have been changes in the geogenic material and the presence of specific anthropogenic materials and pedofeatures. We proceed to describe the microfacies in relation with the three phases of the ritual activity described in the main text. Unfortunately, due to the sampling strategy, phase two and three have not been identified in thin section.

**Phase 0: prior to the animal sacrifices**

***Microfacies type 1: Moderately sorted sands with anthropogenic materials***

Coarse mineral material is composed of silt to fine sand-size smooth angular quartz (30%); coarse sand-size smooth angular quartz (10%); coarse gravel-size to coarse sand-size smooth rounded quartzite (5%); coarse gravel-size smooth rounded slate (2%); and silt-size muscovite (2%). Coarse anthropogenic materials are composed of abundant silt to sand-size charcoal (10%), and few sand-size rounded bone fragments (2%). These components show a coarse monic c/f related distribution. Porosity is mainly composed of simple packing voids. Besides, there are pseudomorphic mouldic voids showing coatings of humified organic matter. This microfacies shows a pellicular to bridged intergrain microstructure and an undifferentiated b-fabric.

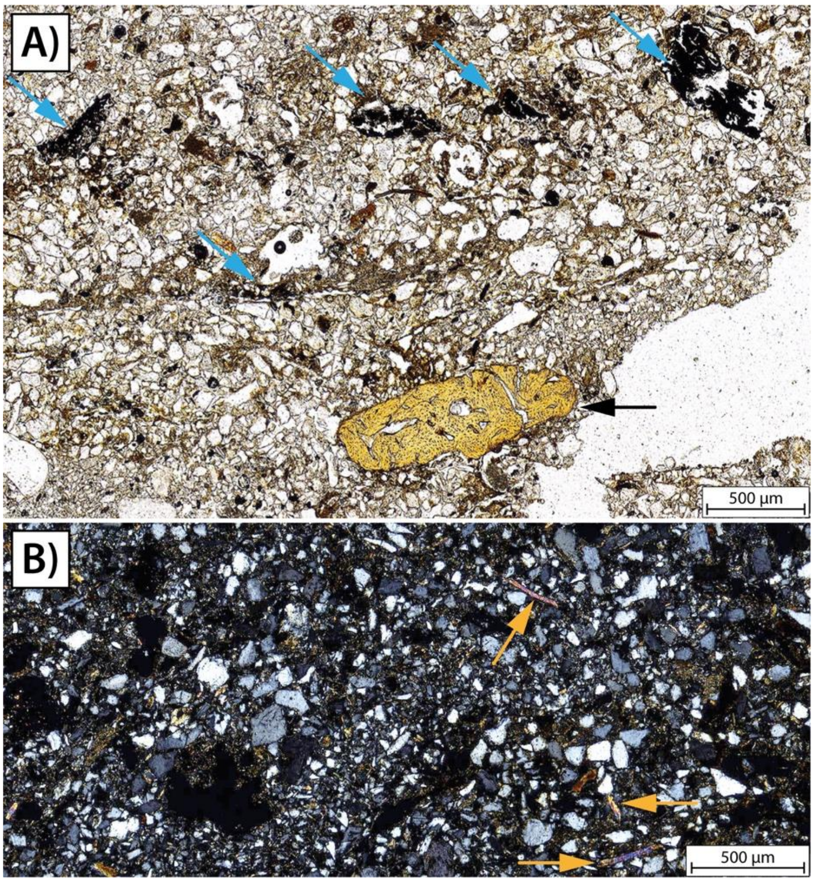


*Figure 4: Microfacies type 1. A) Moderately sorted showing anthropogenic materials, like charcoals (blue arrows) and bones (black arrow); B) Mineralogically, this microfacies is composed of quartz and muscovite (orange arrows)*

***Microfacies type 2: Clayey beaten floor with redoximorphic features***

Coarse mineral fraction is composed of silt to fine sand-size subangular quartz (40%); sand-size subangular smooth quartz (10%); fine sand-size subrounded plagioclase (2%); silt to fine sand-size muscovite (10%); silt to fine sand-size biotite (2%); and sand-size subrounded quartzite (2%). Coarse anthropogenic material is absent. Fine material is composed of reddish orange (PPL) to golden yellow (XPL) clay. This microfacies shows a single spaced porphyric c/f-related distribution, a cross-striated to porostriated b-fabric, and a massive microstructure. Porosity shows abundant pseudomorphic mouldic voids after organic matter decay. As pedofeatures, there are impregnative Fe-Mn hypocoatings identified as *bog iron*. These are optically isotropic Fe oxides and

zones of banded radiating acicular goethite needles on void walls, ferrihydrite nodules, and pyrite framboids. Rotation features as circular or arcuate alignment of particles around grains are common.

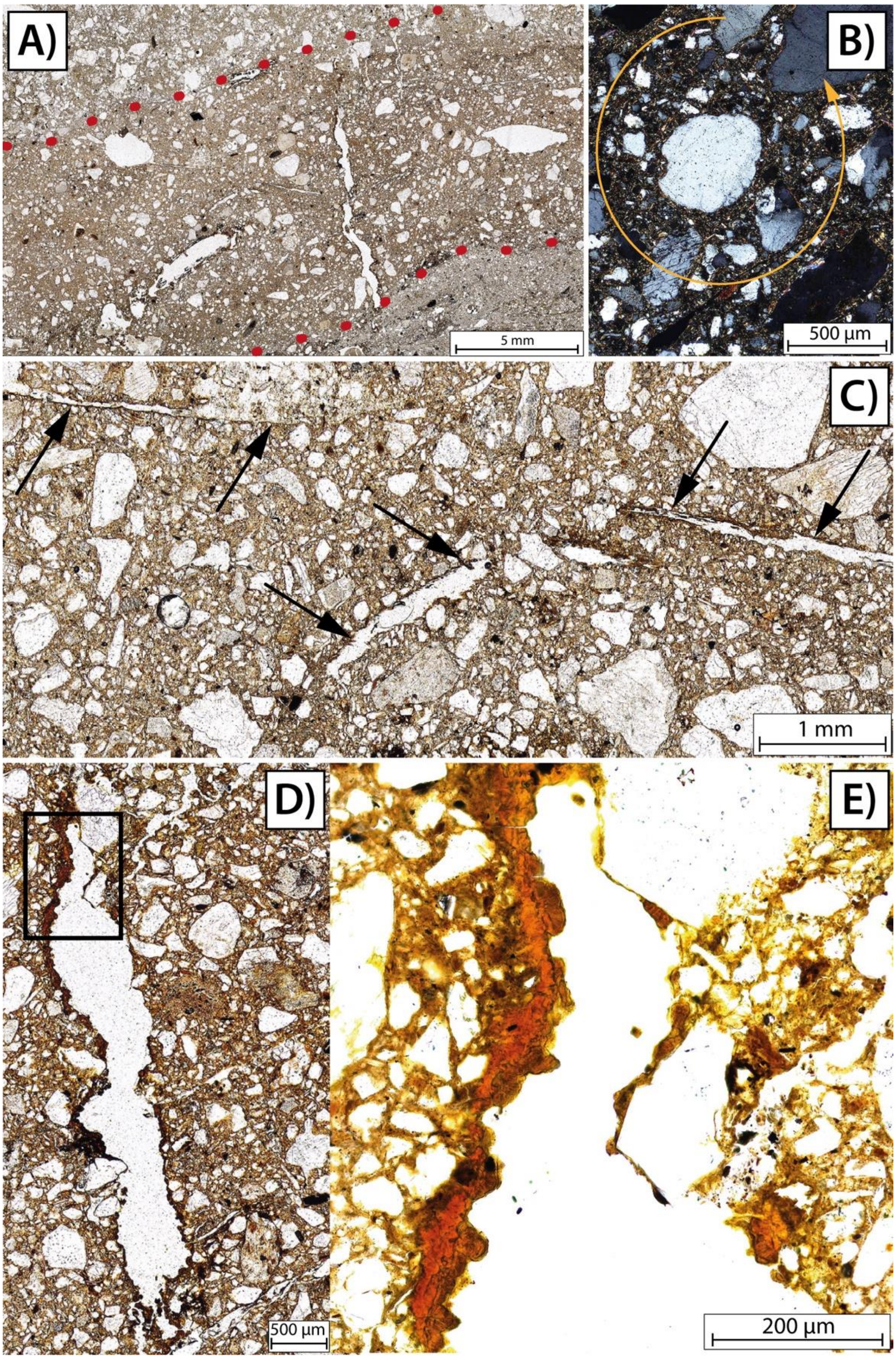


*Figure 5: Microfacies type 2. A) The clayey beaten floor shows a sharp contact with the under- and overlying sediments (red dotted line); B) Rotation features around a quartz grain C) This beaten floor was made by adding chaff as organic binder. After decay, pseudomorphic mouldic voids were formed (black arrows); D) This floor shows Fe/Mn hypocoatings of bog iron produced by waterlogging, water infiltration, and changes in redox state. The black square indicates the detail of e); E) Detail of the Fe/Mn hypocoatings, showing optically isotropic Fe oxides with radial acicular goethite crystals and ferrihydrite nodules*

## Phase 1: first animal sacrifices in the courtyard

***Microfacies type 3. Poorly sorted sandy clay with redoximorphic features and bones***

Coarse mineral fraction is composed of silt to coarse sand-size subrounded quartz (30%); fine sand-size subrounded plagioclase (2%); silt to fine sand-size muscovite (10%); silt to fine sand-size biotite (2%); and fine to coarse sand-size subrounded quartzite (2%). Coarse anthropogenic fraction is composed of few fragments of fine-sand size char and charred organic matter fragments, bones and pottery. Fine material is composed of pale yellow (PPL) to golden yellow (XPL) clay. This microfacies shows a single spaced porphyric c/f-related distribution, a cross-striated b-fabric, and a massive microstructure. Porosity shows abundant polyconcave voids showing a horizontal parallel preferential distribution. As pedofeatures, there are impregnative Fe-Mn oxide nodules and hypocoatings identified as *bog iron*. These are optically isotropic Fe oxides and zones of banded radiating acicular goethite needles on void walls, ferrihydrite nodules, and pyrite framboids. Few weathered phosphatic (horse?) coprolites have been identified. They locally show fibrous structure, phytoliths and organic tissues.

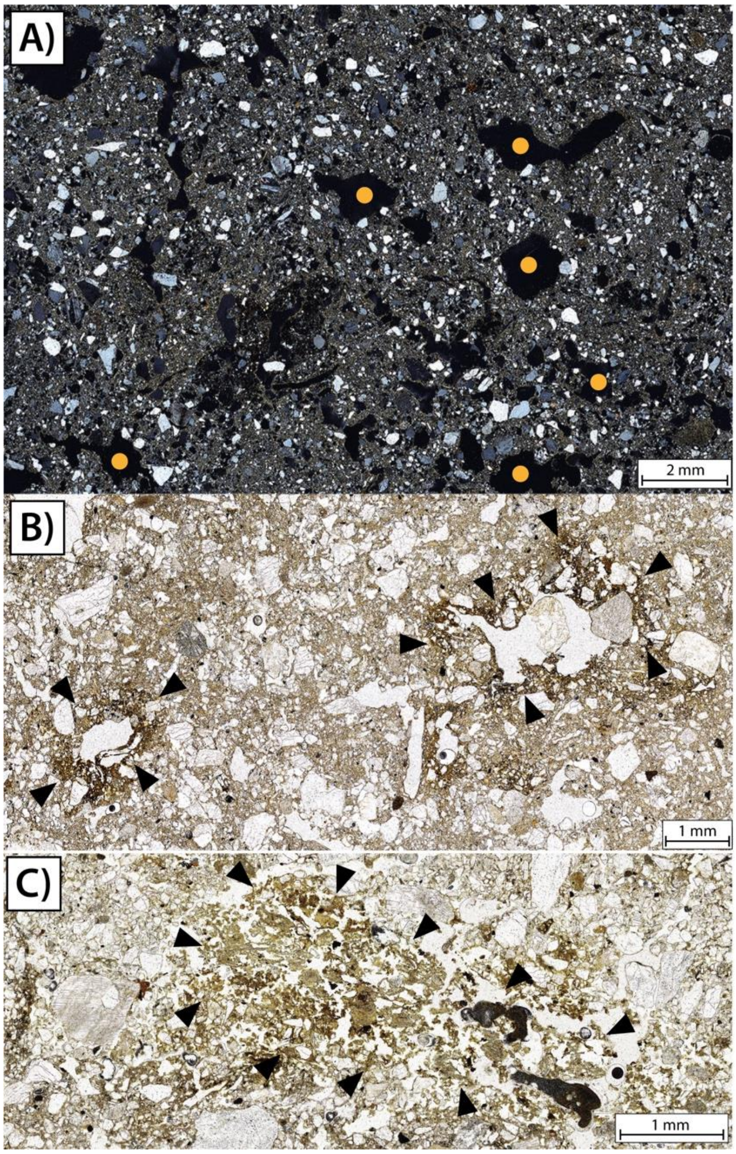


*Figure 6: Microfacies type 3. A) Polyconcave voids are abundant in the sedimentary matrix of the mass sacrifice context, indicating trampling in moist conditions (orange dots); B) Fe/Mn nodules and hypocoatings, indicating waterlogging (black triangles); C) Weathered phosphatic coprolite showing a fibrous microstructure, anatomically connected phytoliths, and organic tissues (black triangles)*

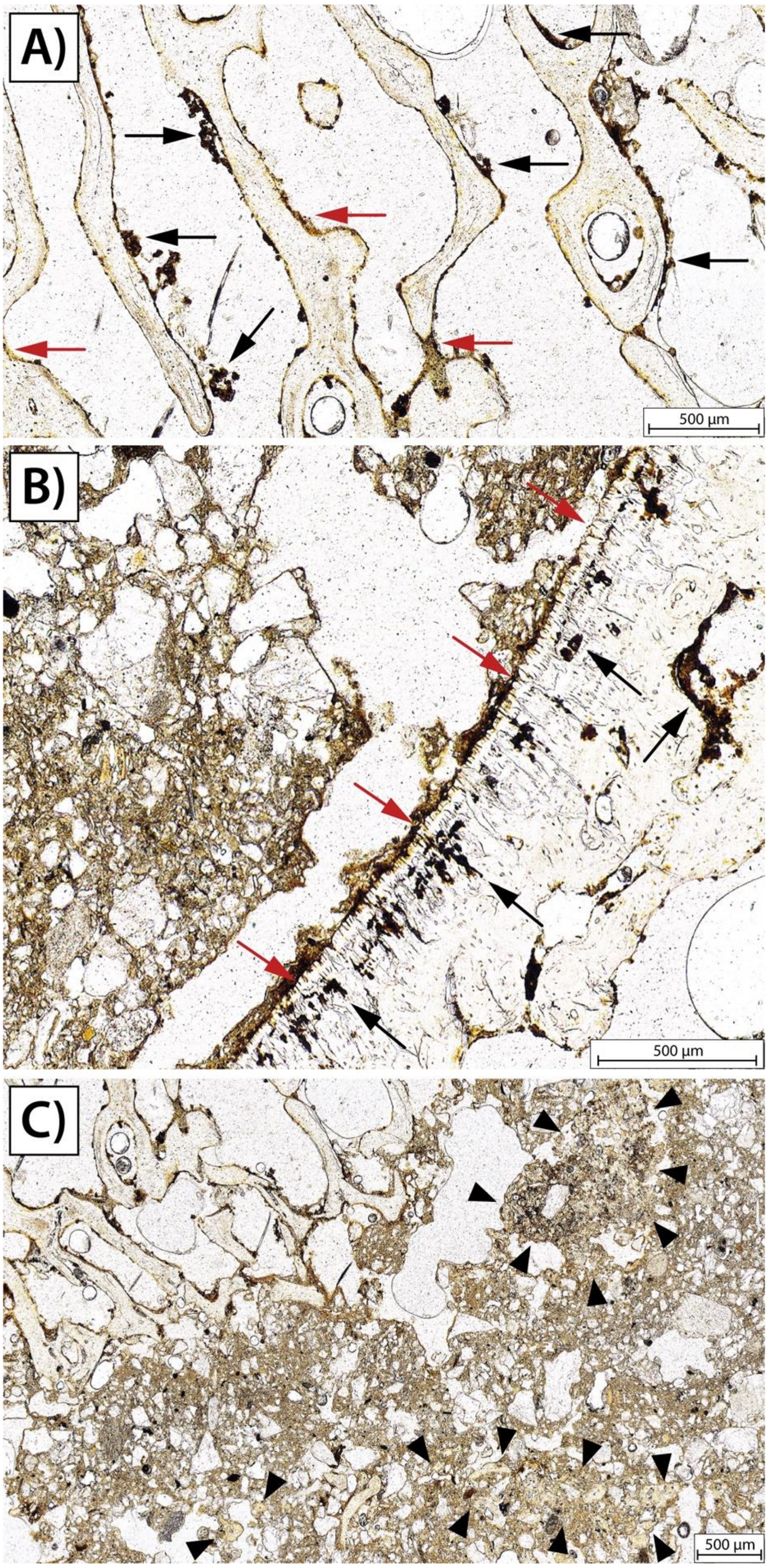


*Figure 7: Microfacies type 3. A) Spongy bone fragment showing silt to fine sand-size aggregates of humified organic matter formed after flesh and tissue decay (black arrows), and laminated silt coatings generated through percolation in waterlogging conditions (red arrows); B) Spongy bone fragment showing serrated edges produced by cyanobacteria after sedimentation and burial of the carcasses (black arrows). Fe/Mn staining indicating postdepositional waterlogging conditions are present (red arrows); c) Phosphate nodules (black arrows) associated to a spongy bone fragment*

**Sediments sealing the last sacrificial deposits of the courtyard**

***Microfacies type 4. Collapse facies composed of mudbrick material***

Coarse mineral material is composed of subrounded silt-size quartz (40%); rounded sand-size quartz (5%); smooth rounded sand-size quartzite (5%); smooth rounded sand-size plagioclase (5%); and few silt-size muscovite crystals (2%) and sand-size fragments of burned mudbrick fragments (1%). Coarse anthropogenic material shows very few charcoal fragments. Fine material is composed of clay, which shows different colors ranging from yellowish-brown to dark brown in PPL, and brown to dark brown being practically isotropic in XPL. This microfacies shows a close porphyric c/f-related distribution, a massive microstructure, and a crystallitic b-fabric. Porosity is composed of pseudomorphic mouldic voids and fissures. As pedofeatures, rotation features and limpid clay intercalations are common.

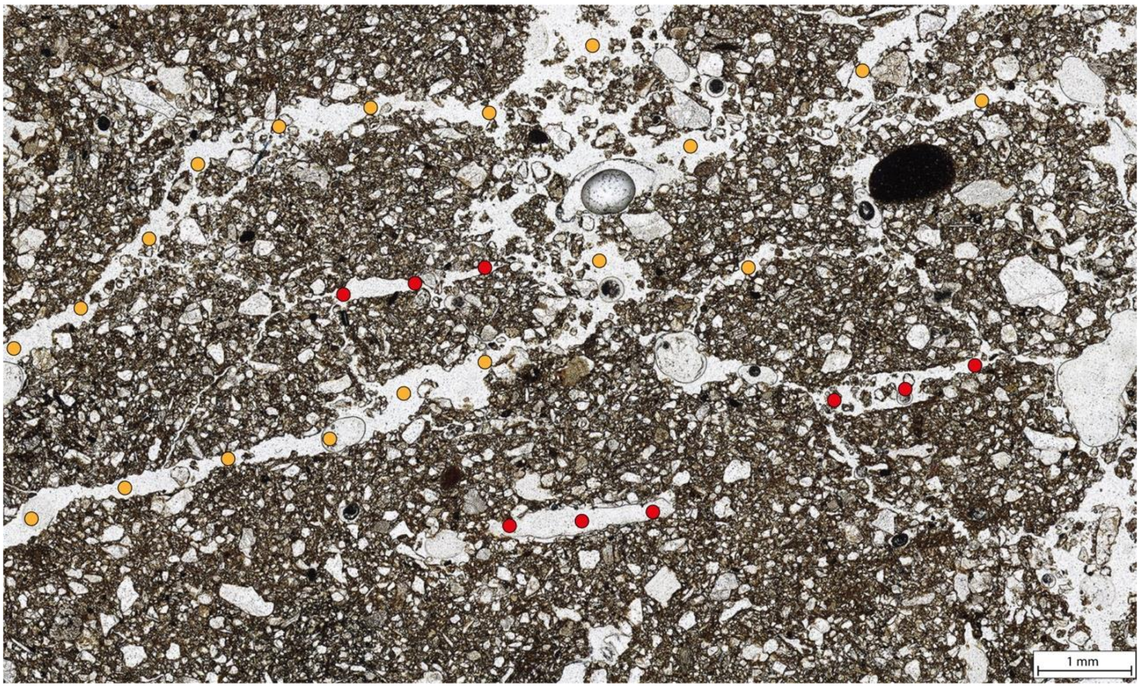


*Figure 8: Microfacies type 4. The sediments sealing the mass sacrificial context show a massive microstructure and a porosity mainly composed of fissures (orange dotted lines) and pseudomorphic mouldic voids after organic matter decay (red dotted lines). This sedimentary material is very similar to the mudbricks used in the building, suggesting that the mass animal sacrifice context was intentionally sealed with a large volume of earth-based construction materials.*

## 3. INTERPRETATION ON THE GENESIS OF THE ARCHAEOLOGICAL SEDIMENTS ASSOCIATED TO THE MASS ANIMAL SACRIFICE CONTEXT AT THE TARTESSIAN SITE OF CASAS DEL TURUÑUELO

The microstratigraphic analysis has allowed us to identify the formation processes and human activities prior, during, and after the mass sacrificial rituals. During the **Phase 0,** before to the sacrificial rituals, **Microfacies type 1**, a quartz sandy material, was found throughout the entire extension of the courtyard. Macroscopically, this sandy sediment shows abundant rounded and discoidal gravels, mainly composed of quartzite. Under the microscope, the mineralogy of this sediment is mainly dominated by silt to sand-size quartz and, proportionally, there are minor amounts of gravel-size quartzite and slate. This mineralogy is compatible with bedload deposits of the Guadiana River, which are

very common in the surroundings of the site. Thus, these materials would have been collected near the site and dumped here, sealing previous occupation phases and the slate slabs of the courtyard, to create a porous horizontal substrate. Human activity is also documented in this occupation phase through the presence of abundant charcoal fragments and bones, perhaps derived from food processing activities, but integrated in the archaeological sediment as part of the constructive infilling.

On top of the sandy sediment, a clayey beaten floor identified as the **Microfacies type 2** was documented. Macroscopically, it is massive and compact, showing a loamy texture and a dark greenish grey color. Under the microscope, this floor is massive and very sandy, showing subangular silt to fine sand quartz in a reddish orange (PPL) to golden yellow (XPL) clayey micromass. These features indicate that this floor was highly trampled in wet conditions[10]. This is also supported by the presence of rotational features in the form of galaxy structures. Experimental research has shown that such structures form in thoroughly kneaded floors under wet conditions[11]. Following the terminology proposed by Gé et al. [12] and later modified by Macphail and Goldberg [10] we have distinguished three different parts of this floor through micromorphology: the passive, reactive, and active layers. The passive layer is the sedimentary material used to construct the floor. This floor shows a sharp basal contact revealing a clear difference between the underlying sandy sediment and the passive layer in terms of composition. In this case, the mineralogy and the mouldic pseudomorphic voids present in this floor points to a local Fluvisol parent material to which chaff was added as an organic binder. Also, we identified the reactive layer, which is generally formed by the mechanical deformation of the passive layer due to trampling. We have identified few rounded sandy silt aggregates resulting from trampling and the disaggregation of the pavement. Finally, the active layer is the topmost part of the floor, resulting from human activity. Typically, under the microscope this last layer is a heterogeneous mix of rounded floor aggregates and residues derived from the human activities developed in that space. In the case of the floor identified in the courtyard, the microfacies 3 can be understood as the active layer. The state of preservation of this floor is generally good in thin section but, at the macroscale, this sediment was mostly documented near the stairs and in spots throughout the courtyard. Thus, when the beaten floor is well-preserved, the slaughtered animals lie on top of it but, in other occasions, the sandy clay sedimentary matrix containing the fauna lies directly on the sandy sediment identified as microfacies type 1, suggesting that the beaten floor was partially reworked and eroded due to the human activities developed in the courtyard.

During the **Phase 1,** associated to the first animal sacrifices carried out in the courtyard, we identify the **Microfacies type 3**. This sandy clay sediment shows abundant anthropogenic materials, like fat-derived char, charcoal, charred organic matter, pottery, and bones of the slaughtered animals. These bones are very indicative of the environmental conditions in which sacrifices took place. The presence of phosphate nodules[13] and the serrated edges of the bone fragments produced by cyanobacteria[14] are indicative of the presence of organic tissues and flesh during the deposition of the animals and in situ decomposition. In this same line, we found abundant aggregates of silt to fine sand-size aggregates of organic matter inside spongy bones. Besides, Fe/Mn features are abundant, both in the bones surface and spongy tissue as staining, indicating postdepositional waterlogging conditions. Also pointing to postdepositional water saturation, silt coatings are common inside the spongy bone tissue. Polyconcave pores showing a horizontal preferred distribution are very common in these sediments. This feature indicate trampling in wet conditions[15], that could

have taken place in the subsequent phases of animal sacrifice identified by taphonomic analysis. Fe/Mn hypocoatings are also present in the micromass, showing optically isotropic Fe oxides with radial acicular goethite crystals and ferrihydrite nodules. These features, known as *bog iron*[16–20], formed postdepositionally in an oxidizing state. Due to water infiltration and percolation to downward sediments, similar Fe/Mn hypocoatings were identified in the previously described beaten floor, indicating contrasting microenvironmental conditions with changes in the redox state due to long-term waterlogging conditions after the deposition of the sacrifices. Finally, highly weathered herbivore coprolites are present in this sediment, showing anatomically connected phytoliths and organic matter inside.

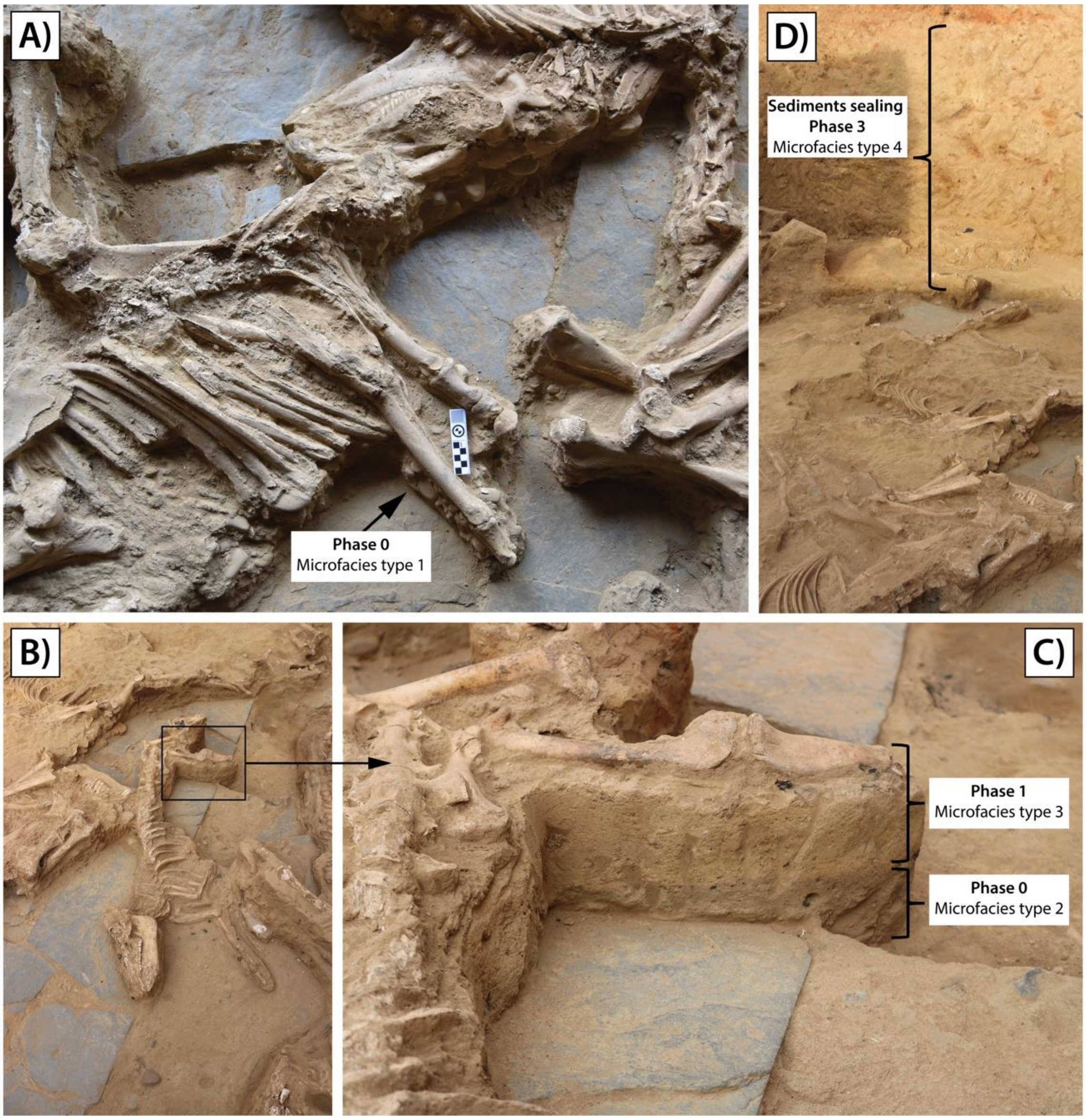


*Figure 9: Correlation of microfacies identified by means of soil micromorphology with the phases identified by taphonomic analysis and the macroscopic archaeological record of the mass animal sacrifice context at Casas del Turuñuelo. A) sandy sediment on top of the slate slabs. Note their common presence below the faunal remains. B) and C) show the clayey beaten floor and the silty sedimentary matrix where most of the animals were found; D) sandy silt sediment sealing the mass animal sacrifice context*

Finally, associated to the **deposits sealing the last animal sacrifices in the courtyard**, we identified the **Microfacies type 4.** This is a silty clay sediment in which the absence

of anthropogenic features (understood as human occupation residues such as bones, charcoal, etc.) is noteworthy. This sedimentary material resembles in composition and microstructure the earth-based construction materials of the site, specifically mudbricks. Thus, it shows a massive microstructure and silt to sand-size quartz dominates the mineralogical assemblage. Again, pseudomorphic mouldic voids formed after the decay of organic matter are very common. We associate this sediment with the intentional dumping and fragmentation of mudbricks. In this process, the dismantling and collapse of the earthen walls of the building sealed the courtyard, favoring the exceptional preservation of the mass sacrificial context.

**References**

1. Courty M-A, Macphail RI, Goldberg P. Soils and Micromorphology in Archaeology. Cambridge: Cambridge University Press; 1989.

2. Macphail RI, Goldberg P. Chapter 27 - Archaeological Materials. In: Stoops G, Marcelino V, Mees F, editors. Interpretation of Micromorphological Features of Soils and Regoliths (Second Edition). Elsevier; 2018. pp. 779–819. doi:10.1016/B978-0-444-63522-8.00027-9

3. Nicosia C, Stoops G, editors. Archaeological Soil and Sediment Micromorphology. John Wiley & Sons, Ltd; 2017. doi:10.1002/9781118941065.index

4. Karkanas P, Goldberg P. Reconstructing Archaeological Sites: Understanding the Geoarchaeological Matrix. Wiley Blackwell; 2019.

5. Stoops G, Marcelino V, Mees F, editors. Interpretation of Micromorphological Features of Soils and Regoliths. Amsterdam: Elsevier; 2010.

6. Stoops G. Guidelines for Analysis and Description of Soil and Regolith Thin Sections. Madison, Wisconsin, USA: Soil Science Society of America Inc.; 2003.

7. Flügel E. Microfacies of carbonate rocks: analysis, interpretation and application. Berlin, Heidelberg: Springer-Verlag; 2004.

8. Courty M-A. Microfacies Analysis Assisting Archaeological Stratigraphy. In: Goldberg P, Holliday VT, Ferring CR, editors. Earth Sciences and Archaeology. Boston, MA: Springer US; 2001. pp. 205–239. Available: http://dx.doi.org/10.1007/978-1-4615-1183-0_8

9. Goldberg P, Miller CE, Schiegl S, Ligouis B, Berna F, Conard NJ, et al. Bedding, hearths, and site maintenance in the Middle Stone Age of Sibudu Cave, KwaZulu-Natal, South Africa. Archaeological and Anthropological Sciences. 2009;1: 95–122. doi:10.1007/s12520-009-0008-1

10. Macphail RI, Goldberg P. Applied Soils and Micromorphology in Archaeology. New York: Cambridge University Press; 2018. doi:10.1017/9780511895562

11. Karkanas P. Microscopic deformation structures in archaeological contexts. Geoarchaeology. 2019;34: 15–29. doi:10.1002/gea.21709

12. Gé T, Courty M-A, Matthews W, Wattez J. Sedimentary formation processes of occupation surfaces. In: Goldberg P, Nash DT, Petraglia MD, editors. Formation processes in archaeological context. Prehistory Press; 1993. pp. 149–163.

13. Karkanas P, Goldberg P. Chapter 12 - Phosphatic Features. In: Stoops G, Marcelino V, Mees F, editors. Interpretation of Micromorphological Features of Soils and Regoliths (Second Edition). Elsevier; 2018. pp. 323–346. doi:10.1016/B978-0-444-63522-8.00012-7

14. Villagran XS, Huisman DJ, Mentzer SM, Miller CE, Jans MM. Bone and Other Skeletal Tissues. In: Nicosia C, Stoops G, editors. Archaeological Soil and Sediment Micromorphology. John Wiley & Sons, Ltd; 2017. pp. 9–38. doi:10.1002/9781118941065.ch1

15. Rentzel P, Nicosia C, Gebhardt A, Brönnimann D, Pümpin C, Ismail-Meyer K. Trampling, Poaching and the Effect of Traffic. In: Nicosia C, Stoops G, editors. Archaeological Soil and Sediment Micromorphology. John Wiley & Sons, Ltd; 2017. pp. 281–297. doi:10.1002/9781118941065.ch30

16. Kaczorek D, Sommer M. Micromorphology, chemistry, and mineralogy of bog iron ores from Poland. CATENA. 2003;54: 393–402. doi:10.1016/S0341-8162(03)00133-4

17. Landuydt CJ. Micromorphology of Iron Minerals from Bog Ores of the Belgian Campine Area. In: Lowell A. Douglas, editor. Developments in Soil Science. Elsevier; 1990. pp. 289–294. Available: http://www.sciencedirect.com/science/article/pii/S0166248108703404

18. Nicosia C, Langohr R, Mees F, Arnoldus-Huyzendveld A, Bruttini J, Cantini F. Medieval Dark Earth in an Active Alluvial Setting from the Uffizi Gallery Complex in Florence, Italy. Geoarchaeology. 2012;27: 105–122. doi:10.1002/gea.21403

19. Stoops G. Sem and light microscopic observations of minerals in bog-ores of the belgian campine. Geoderma. 1983;30: 179–186. doi:10.1016/0016-7061(83)90065-4

20. Vepraskas MJ, Lindbo DL, Stolt MH. Chapter 15 - Redoximorphic Features. In: Stoops G, Marcelino V, Mees F, editors. Interpretation of Micromorphological Features of Soils and Regoliths (Second Edition). Elsevier; 2018. pp. 425–445. doi:10.1016/B978-0-444-63522-8.00015-2
